# Ornithine Lipids in *Burkholderia* spp. Pathogenicity

**DOI:** 10.3389/fmolb.2020.610932

**Published:** 2021-01-05

**Authors:** Luz América Córdoba-Castro, Rosalba Salgado-Morales, Martha Torres, Lourdes Martínez-Aguilar, Luis Lozano, Miguel Ángel Vences-Guzmán, Ziqiang Guan, Edgar Dantán-González, Mario Serrano, Christian Sohlenkamp

**Affiliations:** ^1^Centro de Ciencias Genómicas, Universidad Nacional Autónoma de México, Cuernavaca, Mexico; ^2^Programa de Doctorado en Ciencias Biomédicas, Universidad Nacional Autónoma de México, Centro de Ciencias Genómicas, Cuernavaca, Mexico; ^3^Centro de Investigación en Biotecnología, Universidad Autónoma del Estado de Morelos, Cuernavaca, Mexico; ^4^Department of Biochemistry, Duke University Medical Center, Durham, NC, United States

**Keywords:** pathogenicity, reactive oxygen species, *Burkholderia cenocepacia*, ornithine lipids, *Robbsia andropogonis*

## Abstract

The genus *Burkholderia* sensu lato is composed of a diverse and metabolically versatile group of bacterial species. One characteristic thought to be unique for the genus *Burkholderia* is the presence of two forms each (with and without 2-hydroxylation) of the membrane lipids phosphatidylethanolamine (PE) and ornithine lipids (OLs). Here, we show that only *Burkholderia* sensu stricto strains constitutively form OLs, whereas all other analyzed strains belonging to the *Burkholderia* sensu lato group constitutively form the two forms of PE, but no OLs. We selected two model bacteria to study the function of OL in *Burkholderia* sensu lato: (1) *Burkholderia cenocepacia* wild-type which constitutively forms OLs and its mutant deficient in the formation of OLs and (2) *Robbsia andropogonis* (formerly *Burkholderia andropogonis*) which does not form OL constitutively, and a derived strain constitutively forming OLs. Both were characterized under free-living conditions and during pathogenic interactions with their respective hosts. The absence of OLs in *B. cenocepacia* slightly affected bacterial growth under specific abiotic stress conditions such as high temperature and low pH. *B. cenocepacia* lacking OLs caused lower mortality in *Galleria mellonella* larvae while *R. andropogonis* constitutively forming OLs triggers an increased formation of reactive oxygen species immediately after infection of maize leaves, suggesting that OLs can have an important role during the activation of the innate immune response of eukaryotes.

## Introduction

One major function of amphiphilic lipids is to form the lipid bilayer of membranes which serve as semipermeable barriers and limit a cell. The best-known examples are glycerophospholipids such as phosphatidylglycerol (PG), phosphatidylethanolamine (PE), cardiolipin (CL), and phosphatidylcholine (PC). However, depending on the class of organism and the growth conditions, other lipids such as cholesterol, hopanoids, sphingolipids, sulpholipids, glycolipids, betaine lipids, or ornithine lipids (OLs), can be present in membranes in different concentrations. Especially prokaryotic membranes have been shown to contain a large diversity of lipids, some of which are only formed by specific groups of bacteria or under specific stress conditions (Geiger et al., 2010; Sohlenkamp and Geiger, 2016). OLs are phosphorus-free acyloxyacyl aminolipids which have been found only in bacteria and are apparently absent in eukaryotes or archaea. Their basic structure is composed of a 3-hydroxylated fatty acid linked by an amide bond to the α-amino group of ornithine and a second fatty acid linked by an ester bond to the 3-hydroxyl group of the first fatty acid (Geiger et al., 2010; Sohlenkamp and Geiger, 2016). These lipids can be formed by the OlsBA acyltransferases originally described in *Sinorhizobium meliloti* (Weissenmayer et al., 2002; Gao et al., 2004) or by the bifunctional acyltransferase OlsF first described in *Serratia proteamaculans* (Vences-Guzmán et al., 2015). The OlsBA pathway is present in several α- and β-proteobacteria, in a few γ-proteobacteria and several actinomycetes. Genes encoding OlsF are present in a few γ-proteobacteria, δ- and ε-proteobacteria and in bacteria belonging to the Cytophaga-Flavobacterium-Bacteroidetes (CFB) group. Based on the analysis of genomic DNA sequences, it has been estimated that about 50% of the bacterial species can form OLs at least under specific growth conditions (Vences-Guzmán et al., 2015; Sohlenkamp and Geiger, 2016). Interestingly, in some bacteria, for example *S. meliloti* or *Pseudomonas* sp., OLs are only formed under phosphate-limiting conditions (Minnikin and Abdolrahimzadeh, 1974; Geiger et al., 1999; López-Lara et al., 2005), while in other bacteria, like many species of the genus *Burkholderia* or in *Rhizobium tropici* CIAT899, OLs are formed constitutively (Rojas-Jiménez et al., 2005; González-Silva et al., 2011). In some examples, the presence of OLs or an increased accumulation of OLs have been related to resistance to abiotic stress conditions or to a function during interactions with eukaryotic hosts like an increased persistence (Kim et al., 2018).

The genus *Burkholderia* sensu lato (s.l.) contains more than 100 diverse and versatile bacterial species, which can be found in different environments such as soil or fresh water, but also frequently in association with a number of eukaryotic hosts including humans, animals (vertebrates and invertebrates), plants or fungi. These bacteria-host interactions can be beneficial, pathogenic or both (Depoorter et al., 2016; Estrada-de Los Santos et al., 2018). In recent years, *Burkholderia* s.l. was divided into several genera and it is currently classified into *Burkholderia* sensu stricto (s.s.), *Paraburkholderia* (Sawana et al., 2014), *Caballeronia* (Dobritsa and Samadpour, 2016), *Robbsia* (Rojas-Rojas et al., 2019) and the recently proposed additional genera *Mycetohabitans* and *Trinickia* (Estrada-de Los Santos et al., 2018).

One characteristic that was thought to be unique and common to the species of the genus *Burkholderia* s.l was the presence of two forms of PE and OLs (Yabuuchi et al., 1992). However, when the membrane lipids of a few strains were examined in detail, OLs were present only in some species of the genus (Palleroni, 2015), but were absent in others such as *B. andropogonis* (now classified as *R. andropogonis*). The latter synthesizes PE and hydroxylated PE (2-OH-PE), but lacks OLs and hydroxylated OL (2-OH-OL). The biological roles played by OLs in the genus *Burkholderia* s.l. are not clear yet. As in other bacterial groups, the presence of hydroxylated OLs might be part of the response to environmental stresses. The amount of hydroxylated lipids (2OH-PE and 2OH-OL) was increased in *B. cepacia* strain NCTC 10661 when exposed to a temperature of 42°C, which can be interpreted as a response to thermal stress (Taylor et al., 1998). *B. cenocepacia* J2315 formed a new hydroxylated OL when the strain was exposed to pH 4.0 (González-Silva et al., 2011). Also, rhamnolipids and OLs from *B. pseudomallei* strain K96243 induced an immune response in goats, inducing IFN-γ required for the expression of secreted cytokines (González-Juarrero et al., 2013). Finally, antibacterial activity against *Bacillus megaterium* and *Escherichia coli* has been ascribed to OLs in *B. gladioli* pv. *agaricicola* strain ICMP 11096 (Elshafie et al., 2017).

In this study, we wanted to understand the function of OLs in the genus *Burkholderia* s.l. First, we studied the membrane lipid compositions of a representative set of bacterial species of this group to find out how widespread the presence of OLs and the hydroxylated forms of PE and OL is. *B. cenocepacia* and *R. andropogonis* were selected as models for the second part of this study. *B. cenocepacia* wild-type forms OLs in a constitutive manner and was compared to a mutant unable to synthesize OLs. *R. andropogonis* does not form OLs when grown in normal complex medium and we compared it to a constructed *R. andropogonis* strain constitutively forming OLs. The absence of OLs affected the growth of *B. cenocepacia* under acid stress conditions and it decreased its virulence in a *Galleria mellonella* L (Lepidoptera: Pyralidae) model. The constitutive presence of OLs in *R. andropogonis* caused a stimulation of the plant innate immune response by increasing the production of reactive oxygen species. Both observations highlight the importance of OLs during the interactions between bacteria and their hosts.

## Materials and Methods

### Bacterial Strains, Plasmids, and Growth Conditions

The bacterial strains and plasmids used in this study are listed in the Supporting Material (Supplementary Table 1). Bacteria were grown in Luria–Bertani broth (LB; 5 g yeast extract, 10 g peptone, 10 g NaCl per liter and adding 1.5 % agar (w/v) for solid medium) or in a minimal medium designed for growth under phosphate-limiting conditions. The composition of the latter was based on M9 medium (Miller, 1972) and sodium citrate medium (Simmons, 1926) (2 g Sodium succinate dibasic hexa-hydrated, 1 g NH_4_Cl, 0.2 g MgSO_4_ × 7H_2_O, 0.5 g NaCl and 0.1 M K-phosphate buffer prepared with K_2_HPO_4_ and KH_2_PO_4_). Bacterial growth was determined by measuring the optical density of the cultures at 620 nm (OD620). When required, antibiotics were added to the medium at the following concentrations: 20 μg/ml tetracycline and 50 μg/ml kanamycin for *R. andropogonis* (LMG2129.pRK404.pET9a and LMG2129.pRK404.pET9a.olsF) and 300 μg/ml chloramphenicol for *B. cenocepacia* mutant NG1.

### Construction of the Phylogenetic Tree

The GET_HOMOLOGUES program (Contreras-Moreira and Vinuesa, 2013) was used to obtain families of orthologs from 43 *Burkholderia* s.l. genomes that were downloaded from the NCBI RefSeq database (Supplementary Table 2). In this analysis, each ortholog family had 43 genes. Subsequently, with the help of the GET_PHYLOMARKERS program (Vinuesa et al., 2018), the families of orthologs were analyzed to obtain optimal markers for phylogenomic reconstruction. Finally, with the PHYML program (Guindon et al., 2010), the phylogenetic tree was constructed based on the concatenated alignments of ortholog gene families using the GTR + G evolutionary model.

### Determination of the Membrane Lipid Composition

The lipid compositions of bacterial strains were determined following labeling with [1-^14^C]acetate (Amersham Biosciences). Cultures (1 ml) of *Burkholderia* s.l. strains were inoculated from precultures grown in the same medium. After addition of 1 μCi of [^14^C]acetate (60 mCi mmol-1) to each culture, the cultures were incubated overnight. Cells were harvested by centrifugation, washed with 500 μl water once, resuspended in 100 μl water, and then lipids were extracted according to Bligh and Dyer (Bligh and Dyer, 1959). Aliquots of the lipid extracts were spotted on high performance TLC silica gel 60 plates (Merck, Poole, UK) and separated in two dimensions using chloroform/methanol/water (16:4:1, v/v/v) as a mobile phase for the first dimension and chloroform/methanol/acetic acid (15:3:2, v/v/v) as a mobile phase for the second dimension (Tahara and Fujiyoshi, 1994). To visualize membrane lipids, developed two-dimensional TLC plates were exposed to autoradiography film (Kodak) or to a PhosphorImager screen (Amersham Biosciences). The individual lipids were quantified using ImageQuant software (Amersham Biosciences) (Vences-Guzmán et al., 2011).

### Construction of a *R. andropogonis* Strain Constitutively Producing Ornithine Lipids

The bifunctional acyltransferase OlsF was expressed in the type strain *R. andropogonis* LMG2129. The plasmid pRK404.pET9a.OlsF containing the *olsF* gene from *S. proteamaculans* was mobilized by conjugal transfer from *E. coli* S17-1 into the recipient strain *R. andropogonis* LMG2129 (Simon et al., 1983). Precultures with an OD620 of 0.5 of donor and recipient cells were harvested by centrifugation and washed twice with LB medium to remove residual antibiotics. Subsequently, the cell suspensions were mixed, dropped onto LB agar plates without antibiotics and incubated overnight at 30°C. The cells were scraped from the plates, suspended, and serial aliquots were plated on sodium citrate agar (Simmons, 1926) supplemented with tetracycline and kanamycin. Incubation continued for 2–5 days to select for the presence of the plasmid in the transconjugants. Transconjugant colonies were transferred to plates with LB medium supplemented with tetracycline and kanamycin and were grown at 30°C for 3 days. Strains were conserved in glycerol at a final concentration of 30% (w/v) and were stored at −80°C.

### Liquid Chromatography/Tandem Mass Spectrometry Analysis of Lipid Samples

The three different *R. andropogonis* strains (LMG2129.pRK404.pET9a.olsF-expressing the acyltransferase OlsF, LMG2129-wild-type, and LMG2129.pRK404.pET9a-empty vector control) were grown to an OD of 1.2 at 620 nm in LB medium or in LB medium with tetracycline in case of the plasmid-harboring strains. Cells were harvested by centrifugation and lipids were extracted according to Bligh and Dyer (Bligh and Dyer, 1959). Normal phase LC-ESI MS of the lipid extracts was performed using an Agilent 1200 Quaternary LC system coupled to a high resolution TripleTOF5600 mass spectrometer (Sciex, Framingham, MA). Chromatographic separation was performed on an Ascentis Silica HPLC column, 5 μm, 25 cm × 2.1 mm (Sigma-Aldrich, St. Louis, MO). Lipids were eluted with mobile phase A, consisting of chloroform/methanol/aqueous ammonium hydroxide (800:195:5, v/v/v), mobile phase B, consisting of chloroform/methanol/water/aqueous ammonium hydroxide (600:340:50:5, v/v/v/v) and mobile phase C, consisting of chloroform/methanol/water/aqueous ammonium hydroxide (450:450:95:5, v/v/v/v), over a 40 min-long run, performed as follows: 100% mobile phase A was held isocratically for 2 min and then linearly increased to 100% mobile phase B over 14 min and held at 100% B for 11 min. The mobile phase composition was then changed to 100% mobile phase C over 3 min and held at 100% C for 3 min, and finally returned to 100% A over 0.5 min and held at 100% A for 5 min. The LC eluent (with a total flow rate of 300 μl/min) was introduced into the ESI source of the high resolution TF5600 mass spectrometer. MS and MS/MS were performed in negative ion mode, with the full-scan spectra being collected in the *m/z* 200–2,000 range. The MS settings are as follows: ion spray voltage (IS) = −4,500 V (negative ion mode), curtain gas (CUR) = 20 psi, ion source gas 1 (GS1) = 20 psi, de-clustering potential (DP) = −55 V, and focusing potential (FP) = −150 V. Nitrogen was used as the collision gas for tandem mass spectrometry (MS/MS) experiments. Data analysis was performed using Analyst TF1.5 software (Sciex, Framingham, MA).

### Identification of Putative Pho Boxes in *Burkholderia* sensu latu

The program INFO-GIBBS (Defrance and van Helden, 2009) was used for the construction of a position specific scoring matrix (PSSM) matrix. Pho box sequences from *Agrobacterium tumefaciens, Sinorhizobium meliloti*, and *Mesorhizobium loti* identified in the promoter sequences of genes involved in the biosynthesis of glycolipid and OL membrane lipids were used (Supplementary Table 3) (Yuan et al., 2006; Geske et al., 2013). The following parameters were used: Matrix length was fixed to 18 bp, the expected number of sites per sequence was one, and the number of motifs to extract was one. As background model the sequences upstream of genes in the *Burkholderiaceae* taxon were used and the Markov order used was one. Then the program MATRIX-SCAN (Turatsinze et al., 2008) was used to search for putative Pho boxes in the regions upstream of the genes encoding OlsB homologs in the genomes of the 43 *Burkholderia* s.l. species analyzed. This search was performed for both strands.

### Growth Experiments Under Abiotic Stress Conditions

Pre-cultures of *R. andropogonis* and *B. cenocepacia* were grown at 30°C at 250 rpm overnight in LB medium supplemented with the respective antibiotics if required. Cells were harvested by centrifugation at 6,000 rpm and washed with 1% NaCl (w/v) solution, except for the salinity tests for which cells were washed in LB medium without salt. The optical density data collection was measured in the Synergy 2.0 Biotek, measuring every 3 h for 24 h, performing 3 independent repetitions. For temperature stress experiments, the cultures were grown in LB medium in 96-well microplates, inoculated with an OD620 of 0.05 and incubated at 30, 37, or 42°C. For acid stress experiments, the cultures were grown in 96-well microplates in LB medium adjusted to pH 4 buffered with 50 mM Homopipes [Homopiperazine-*N,N*′-bis-2-(ethanesulfonic acid)] or to pH 7 with 50 mM Pipes [piperazine-*N,N*′-bis(2-ethanesulfonic acid)]. Cultures were inoculated at an OD620 of 0.05 and incubated at 30°C with medium shaking. For salinity stress experiments, the cultures were grown in 96-well microplates in LB medium with salt concentrations of 0.05, 0.5, and 1 M NaCl. Cultures were inoculated at an OD620 of 0.05 and incubated at 30°C with medium shaking.

### *B. cenocepacia* Virulence Assays Using *Galleria mellonella* Larvae

The virulence of the *B. cenocepacia* strains was evaluated in *G. mellonella* larvae. Starting from bacterial cultures grown to an OD620 of 1, serial dilutions were made in 10 mM MgSO_4_ that corresponded to 3 × 10^7^, 3 × 10^6^, 3 × 10^5^, and 2 × 10^4^ CFU/ml. As negative controls, a simple puncture of the larvae, the injection of saline solution, or the injection of *E. coli* DH5α (Hanahan, 1983) at 3 × 10^6^ CFU/ml were used. This strain is innocuous for *G. mellonella* (Alghoribi et al., 2014; Jonsson et al., 2017). Assays were carried out using the injection method with *G. mellonella* larvae in the sixth stage and 10 μl bacterial suspensions were injected into the dorsal region of the third anterior abdominal segment of the larvae using insulin syringe of 31G (gauge). Each bacterial suspension was tested using 10 insect larvae placed individually in 55 mm petri dishes without diet and incubated at 30°C. The mortality was evaluated every 24 h for 5 days after injection. Five independent experiments were performed. For statistical testing, experimental data (*n* = 50) were plotted using the Kaplan-Meier method and differences in survival were calculated by using the log-rank test with a *p* ≤ 0.05 indicating statistical significance. The statistical analyses were performed using GraphPad Prism, version 8.4.3 (GraphPad Software Inc., San Diego, CA, USA).

### Infiltration of Maize Plants With *R. andropogonis* Strains

*R. andropogonis* assays were carried out in corn plants using a native maize variety (“criollo de Hidalgo”). Seed sterilization and germination were performed as previously described (Matus-Acuña et al., 2018), and sterilized seeds were incubated for 48 h at 30°C in the dark. Subsequently, germinated seedlings were transplanted to pots containing sterile vermiculite and were grown under greenhouse conditions irrigating every 3 or 4 days with water or Fahraeus solution for 40 days before infection with the bacterial strains (Fahraeus, 1957). Maize leaves were infiltrated on the bottom of the leaves with a 1 ml needleless syringe containing a 1 × 10^5^ CFU/ml bacterial suspension (corresponding to an OD620 of about 0.02) of the wild-type strain LMG2129 or the transconjugants (LMG2129.pRK404.pET9a, LMG2129.pRK404.pET9a.olsF). As mock control a 10 mM MgCl_2_ solution was used. Approximately 10 μl were infiltrated into the leaf at each site. Forty healthy 40 day-old maize plants were used. Ten potted plants were inoculated with each isolate and the development of symptoms was followed for 15 days. The bacteria were isolated using a methodology previously described (Katagiri et al., 2002). Briefly, treated maize leaves were macerated with pistil, washed with 10 mM MgCl_2_ twice and finally resuspended in LB medium. The suspension was serially diluted, plated on LB medium and incubated at 30°C for 72 h. Colonies were counted and colony forming units determined. For statistical analyses the one-tailed *t*-test was used with a *p* ≤ 0.05 indicating statistical significance. Statistical analyses were performed using GraphPad Prism, version 8.4.3 (GraphPad Software Inc., San Diego, CA, USA).

### Detection of Reactive Oxygen Species (ROS)

ROS were detected using the fluorescent probe 2′,7′-Dichlorodihydrofluorescein diacetate (DCFH-DA; Sigma-Aldrich, www.sigmaaldrich.com) as previously described (L'Haridon et al., 2011). Leaves were rapidly rinsed in DCFH-DA medium and observed under UV light with a LEICA DMR fluorescence microscope (Leica, www.leica.com). Microscope images were saved as TIFF files and processed for quantification of the pixels with Image J version 1.51 (NIH). For statistical analyses the one-tailed *t*-test was used with a *p* ≤ 0.05 indicating statistical significance. Statistical analyses were performed using GraphPad Prism, version 8.4.3 (GraphPad Software Inc., San Diego, CA, USA).

## Results

### *Burkholderia* sensu lato Strains and Their Membrane Lipid Compositions: Only sensu strictu Strains Form OL Constitutively

**Figure 1:**
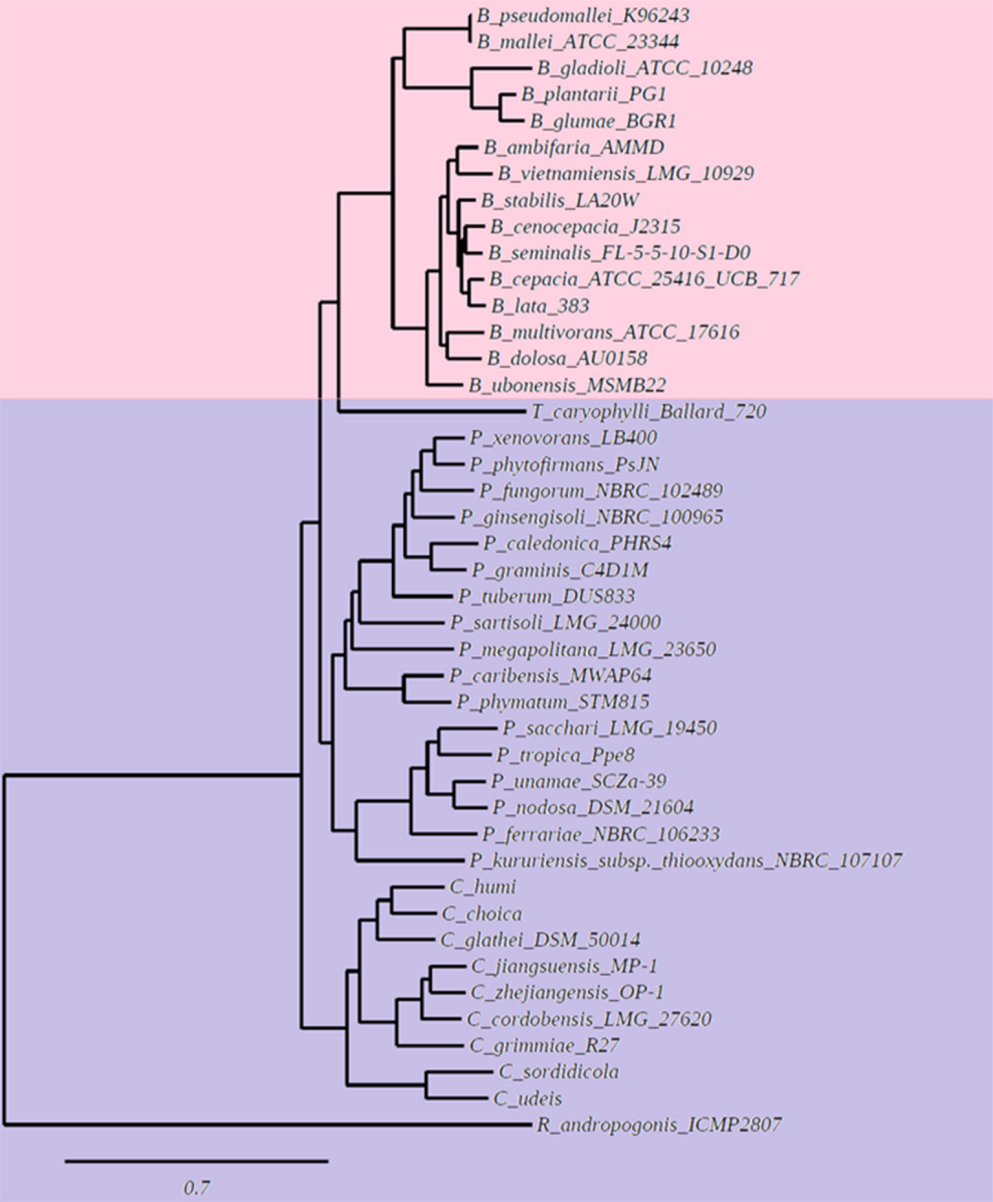
Phylogenetic tree based on a pangenome analysis of *Burkholderiales*. The first clade (indicated with pink color) groups species of the genus *Burkholderia* sensu stricto (s.s.) that synthesize unmodified and modified OLs when grown in complex LB medium. The second clade (marked in purple color) groups species of the genera *Trinickia, Paraburkholderia, Caballeronia*, and *Robbsia*, that do not synthesize OLs when grown in complex LB medium.

**Figure 2:**
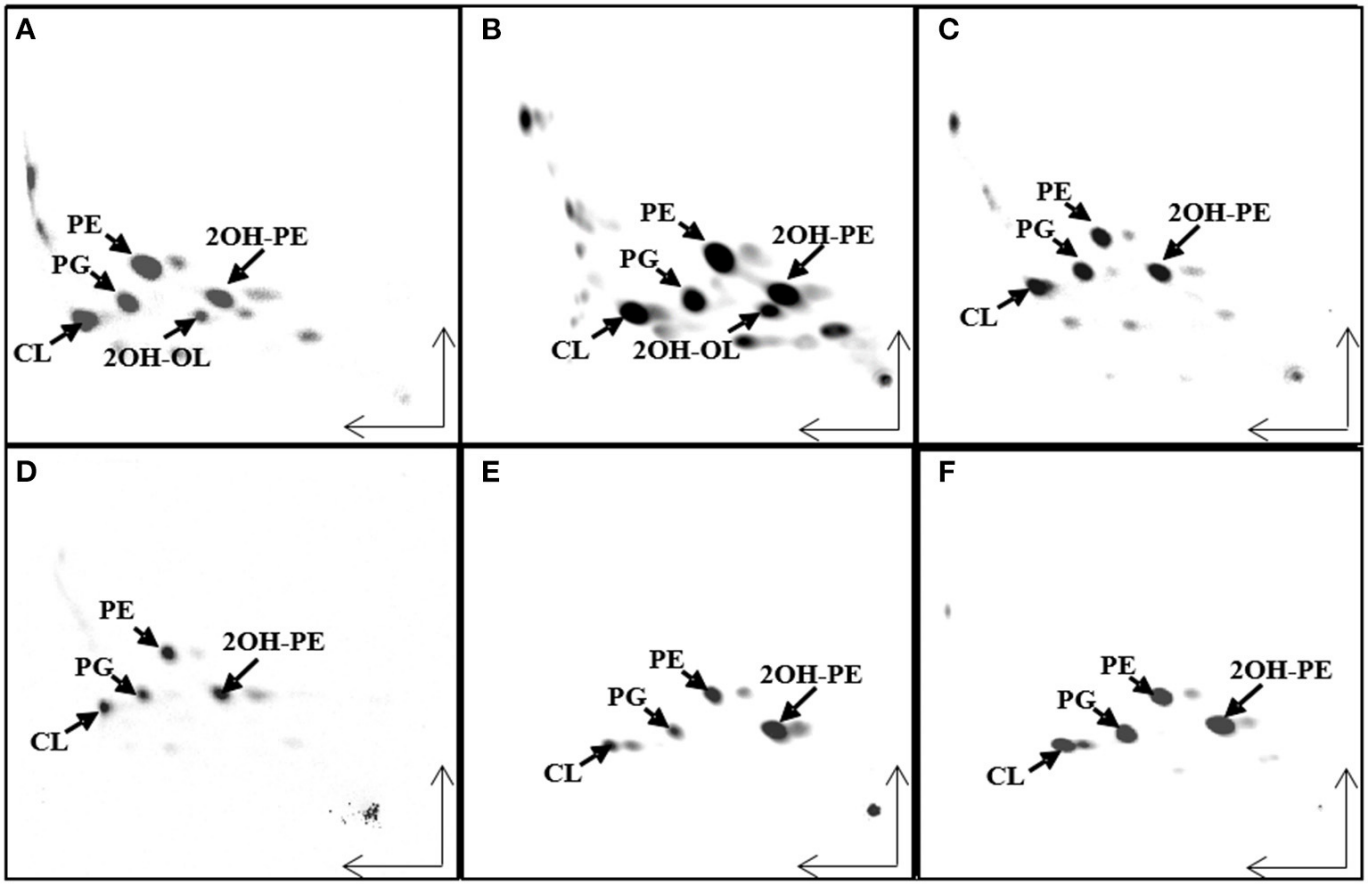
Ornithine lipid (OL) synthesis is constitutive in strains belonging to the genus *Burkholderia* sensu stricto (s.s), but not in the other analyzed strains. Separation of [^14^C]acetate-labeled lipids by two dimensional thin-layer chromatography (TLC) from different *Burkholderia* sensu latu (s.l) strains grown in LB medium at 30°C. **(A)** *Burkholderia cenocepacia* J2315, **(B)** *B. dolosa*, **(C)** *Paraburkholderia sortisoli*, **(D)** *P. xenovorans*, **(E)** *Caballeronia glathei*, and **(F)** *Robbsia andropogonis*. CL, cardiolipin; PG, phosphatidylglycerol; PE, phosphatidylethanolamine; 2OH-PE, hydroxylated phosphatidylethanolamine; OL, unmodified ornithine lipid; 2OH-OL, ornithine lipid 2-hydroxylated within ester-bound fatty acid.

We wanted to study if there is a correlation between the membrane lipid composition of the species and their phylogenic positions within the *Burkholderia* sensu lato (s.l.) supergenus. An analysis performed with the genomes of 43 *Burkholderia* s.l. species indicated that 780 gene families, representing groups of orthologs, were present in each of the 43 genomes. Next, using the Get Phylomarkers software (Vinuesa et al., 2018), 411 families of orthologs were considered optimal markers for a phylogenomic reconstruction of the *Burkholderia* s.l. supergenus. The phylogeny showed that *Burkholderia* s.l. was separated into five different lineages (Figure 1). These corresponded to *Burkholderia* s.s., *Trinickia, Paraburkholderia, Caballeronia*, and *Robbsia*. We analyzed the membrane lipid composition of 35 *Burkholderia* s.l. strains to which we had access in the laboratory when they were grown in complex medium. Liquid cultures of the selected strains were grown, lipids were labeled with [^14^C]acetate, extracted and separated by two dimensional thin-layer chromatography (Figure 2, six representative strains are shown). In all strains, the lipids phosphatidylglycerol (PG), cardiolipin (CL), phosphatidylethanolamine (PE), and 2-hydroxylated PE (2OH-PE) were detected. However, under the evaluated growth condition OLs be it unmodified and/or modified were only detected in the strains of the genus *Burkholderia* s.s. (Figures 2A,B). The species included in the *Burkholderia* s.s. clade (indicated in pink, Figure 1), are generally species that correspond to pathogens of humans, plants or animals (Depoorter et al., 2016). The clade that groups the genera *Trinickia, Paraburkholderia, Caballeronia*, and *Robbsia* (marked in purple, Figure 1) includes species that do not synthesize OLs when grown in complex medium, and several of these bacteria are plant beneficial and environmental bacteria, or can be involved in an N_2_-fixing symbiosis with legume plants (Estrada-de Los Santos et al., 2018). An exception is the genus *Robbsia* which is formed by plant pathogens.

**Figure 3:**
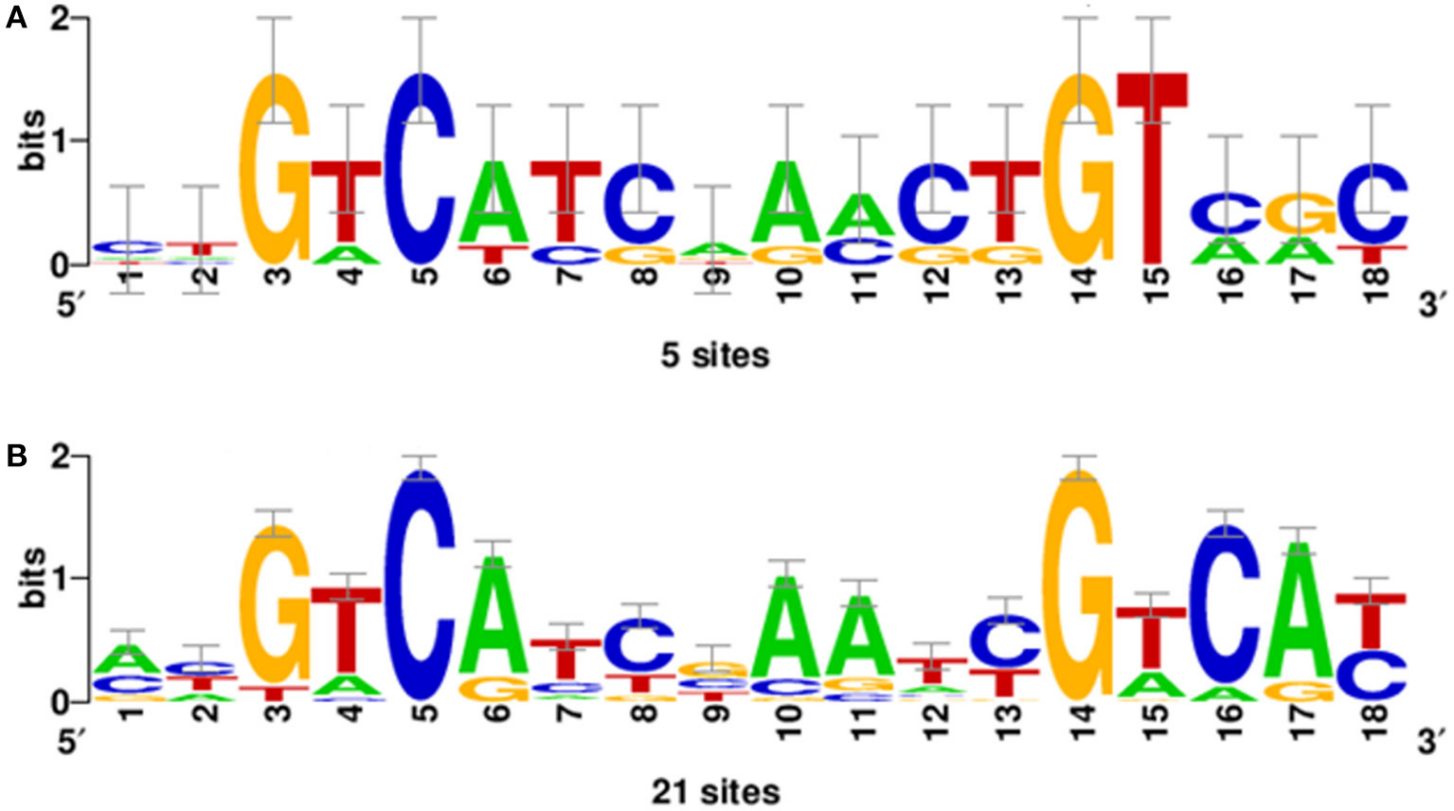
Position-specific scoring matrix (PSSM) of Pho boxes obtained using the INFO-GIBBS program. **(A)** The PSSM derived from the sequences of Pho boxes of *A. tumefaciens, S. meliloti*, and *M. loti* present in the promoter sequences of the genes involved in the lipid biosynthesis of glycolipids and OLs (Supplementary Table 3). **(B)** PSSM obtained of putative Pho boxes from *Burkholderiales* (Supplementary Table 4) that constitutively synthesize OLs or not.

Bacterial species belonging to the genus *Burkholderia* form OLs by the OlsBA pathway. The presence of a gene encoding an OlsB homolog is considered a good indicator for the capacity of the bacteria to form OLs (Geiger et al., 2010). We wanted to know if the absence of OLs in the species classified as *Paraburkholderia, Caballeronia, Robbsia, Mycetohabitans*, and *Trinickia* was caused by the absence of a gene encoding an OlsB homolog or if it was due to a difference in gene regulation. We searched the genomes of *Paraburkholderia, Caballeronia, Robbsia, Mycetohabitans*, and *Trinickia* species for genes encoding homologs of the *N*-acyltransferase OlsB (Bcal1281) responsible for the first step in OL synthesis in *B. cenocepacia* (González-Silva et al., 2011). We found that all analyzed species possessed a gene coding for an OlsB homolog in their genome and that the genomic context around the respective genes was usually conserved in *Burkholderia* s.l. (data no shown). Bacterial species such as *Sinorhizobium meliloti, Rhodobacter sphaeroides, Pseudomonas fluorescens, Pseudomonas diminuta, Desulfovibrio alaskensis, Serratia proteamaculans*, and *Vibrio cholerae* do not form OL when the bacteria are grown in complex media (which are usually rich in phosphate), but induce the synthesis of OLs or/and other phosphorus-free lipids under phosphate-limiting growth conditions and replace some of their membrane phospholipids (Minnikin and Abdolrahimzadeh, 1974; Benning et al., 1995; Geiger et al., 1999; Lewenza et al., 2011; Bosak et al., 2016; Barbosa et al., 2018). In some of these bacteria, gene expression in response to phosphate limitation is known to be regulated by the two-component system PhoBR. At low phosphate concentrations, the response regulator PhoB is phosphorylated and binds to a highly conserved nucleotide motif called the Pho box in the promoter region of regulated genes (Geske et al., 2013). We would expect to find Pho boxes preceding the homologs of *olsB* genes if the synthesis of OLs can be induced at low phosphate concentrations and is regulated by PhoB. Using the sequences of identified Pho boxes from *Agrobacterium tumefaciens, S. meliloti, and Mesorhizobium loti* (Yuan et al., 2006; Geske et al., 2013) present in the promoter sequences of the genes encoding enzymes involved in the biosynthesis of glycolipids and OLs formed under conditions of phosphate limitation (Supplementary Table 3), a consensus sequence was obtained (Figure 3A). The genomes of 43 *Burkholderia* s.l. species were searched for the presence of Pho boxes upstream of the genes encoding OlsB homologs. Putative Pho boxes were detected in 21 of the analyzed genomes (Supplementary Table 4) and corresponded to fourteen species of the genus *Paraburkholderia*, two species of the genus *Caballeronia* (both genera do not constitutively synthesize OLs), and five species corresponding to the genus *Burkholderia* s.s. that constitutively synthesize OLs. Due to the evolutionary distance between α-proteobacteria and β-proteobacteria it is possible that we missed the Pho boxes in some genomes, but alternatively, OL formation might be regulated differently in these species. Using the putative Pho boxes of *Burkholderiales* (Supplementary Table 4), a new matrix was obtained (Figure 3B). When searching with this new matrix, putative Pho boxes were detected in the upstream regions of the genes encoding OlsB homologs in all 43 *Burkholderia* s.l genomes (data not shown).

### OlsF Expression Causes Constitutive OL Formation in *Robbsia andropogonis*

OL synthesis is constitutive in the *Burkholderia* s.s., but probably inducible in the other genera forming part of *Burkholderia* s.l., and an open question is if the presence or absence of OL affects the abiotic stress resistance of the bacteria and if it affects how the bacteria interact or how they are perceived by their eukaryotic hosts. Therefore, we wanted to study two pairs of strains either forming OLs or not, under abiotic stress conditions and during interactions with their eukaryotic hosts: (1) *B. cenocepacia* J2315 (Vandamme et al., 2003), which forms OL constitutively and its corresponding mutant deficient in OL formation NG1 (González-Silva et al., 2011) and (2) *R. andropogonis* (formerly *B. andropogonis*) which does not form OL constitutively and *R. andropogonis* constitutively forming OLs due to the presence of a plasmid harboring the gene *olsF* from *S. proteamaculans* (Vences-Guzmán et al., 2015).

**Figure 4:**
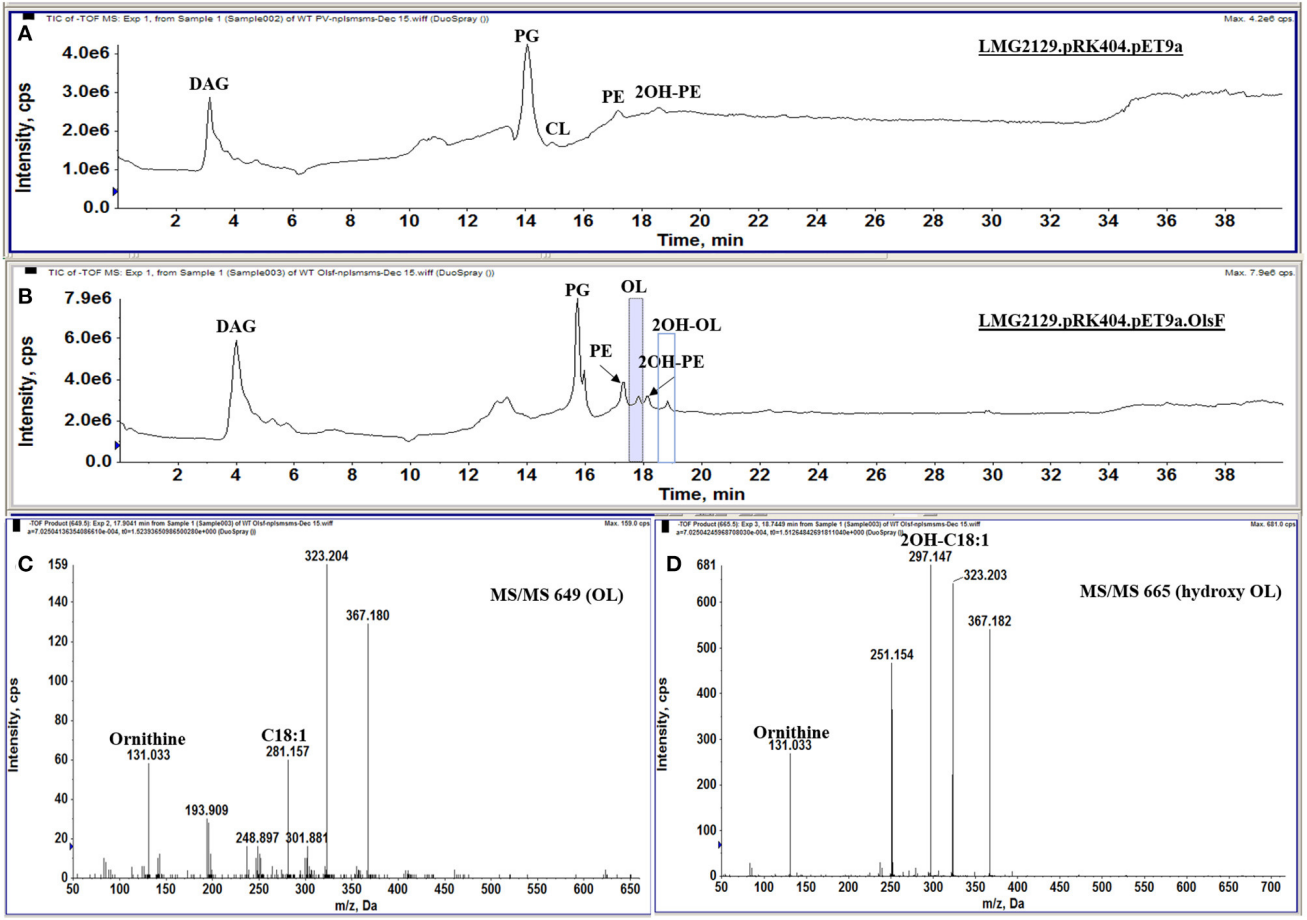
*R. andropogonis* LMG2129 expressing OlsF forms OLs and 2OH-OLs. Shown are total ion chromatograms of the LC-MS analysis of total lipid extracts from **(A)** *R. andropogonis* LMG2129.pRK404.pET9a, and **(B)** *R. andropogonis* LMG2129.pRK404.pET9a.olsF. **(C)** Negative ion collision-induced dissociation mass spectra of [M-H]—ions at *m/z* 649 identifying OL and **(D)** *m/z* 665 showing that it is 2OH-OLs. The masses of major fragment ions indicate that the hydroxyl group is located in the secondary fatty acyl chain.

*R. andropogonis* contains both forms of PE but no OLs when grown in complex medium (Figure 2F). To create a *R. andropogonis* strain constitutively forming OL, the bifunctional acyltranferase OlsF from *S. proteamaculans* (Vences-Guzmán et al., 2015) was expressed in the type strain *R. andropogonis* LMG2129 (Gillis et al., 1995). When analyzing the lipid composition of the OlsF-expressing strain using LC-MS, we observed the formation of two new lipids that were absent in the vector control strain (Figures 4A,B) and the lipid profile of the vector control strain is similar to the wild-type strain. These new lipids were identified as unmodified and hydroxylated OLs (Figures 4C,D), with the [M-H]^−^ ions of their major species being observed at *m/z* 649.5 and 665.5, respectively. A comparison of the MS/MS spectra of *m/z* 649.5 and 665.5 (Figures 4C,D) indicates that the hydroxylation is located within the secondary fatty acyl chain. Specifically, the carboxylic anion of C18:1 fatty acid is observed at *m/z* 281 (Figure 4C), and the carboxylic anion of hydroxylated C18:1 fatty acid is observed at *m/z* 297 (Figure 4D). This result suggests that the hydroxylase responsible for 2-hydroxylation of OL is constitutively expressed whereas OL synthesis is inducible in the wild-type strain.

### The Absence of OLs Slows the Growth of *B. cenocepacia* Under Acid Stress Conditions

Earlier reports had shown that *B. cepacia* induces OL formation under conditions of heat stress (Taylor et al., 1998). An importance of the presence of OL for heat and acid stress resistance had been also described in *R. tropici* (Rojas-Jiménez et al., 2005; Vences-Guzmán et al., 2011). We wondered if the absence of OL in *B. cenocepacia* NG1 (González-Silva et al., 2011) would negatively affect the stress tolerance of this strain in comparison with its respective wild-type J2315 or if the *R. andropogonis* strain constitutively forming OL would be more resistant to abiotic stress than the corresponding wild-type. Strains were exposed to various abiotic stresses such as variations in temperature (30, 37, or 42°C), in acidity (pH 4 or pH 7) or osmotic stress conditions (0.05 M, 0.5 M, or 1 M NaCl).

**Figure 5:**
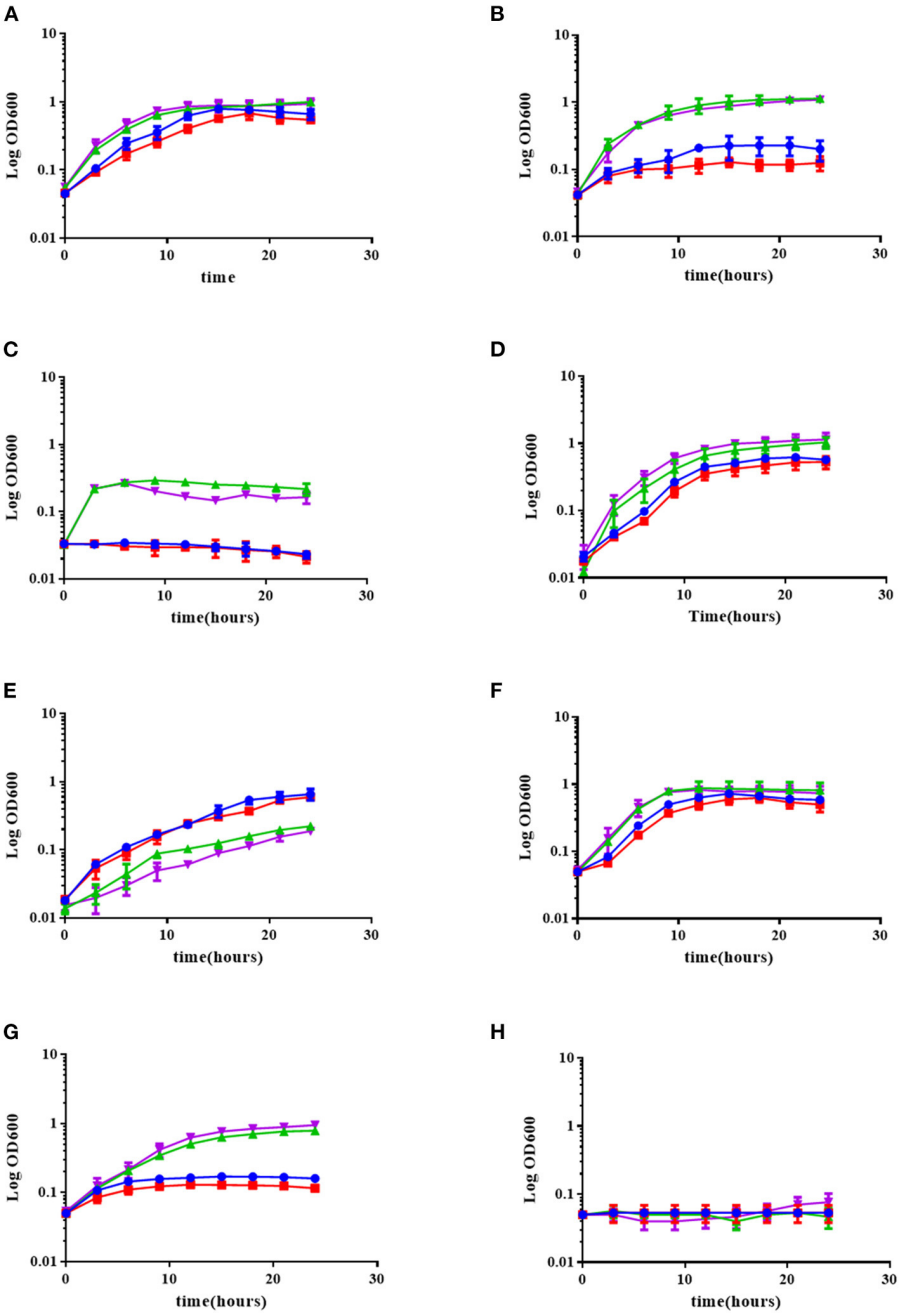
The absence of OLs in the *B. cenocepacia* mutant deficient in *olsB* slightly affects its growth under low pH and temperature stress conditions, but the presence of OLs in *R. andropogonis* does not increase tolerance to environmental stress conditions. The strains were grown in complex LB medium at 30°C **(A)**, 37°C **(B)**, or 42°C **(C)**, or in complex LB medium adjusted to pH 7.0 **(D)**, or pH 4.0 **(E)**, and in complex LB medium supplemented with 0.05 M NaCl **(F)**, 0.5 M NaCl **(G)**, or 1 M NaCl **(H)** at 30°C. Growth kinetics were determined in a Synergy 2.0 Biotek, with medium shaking in 96-well microplates measuring every 3 h for 24 h, performing three independent repetitions. ▲ (green triangle up) *B. cenocepacia* J2315, ▼ (purple triangle down) *B. cenocepacia* NG1, • (blue circle) *R. andropogonis* LMG2129.pRK.pET9a, ■ (red square) *R. andropogonis* LMG2129.pRK.pET9a.olsF.

With respect to the temperature stress experiments, we observed that *R. andropogonis* strains grew best at 30°C (Figure 5A), and their growth was drastically affected at 37°C. The *R. andropogonis* strain constitutively forming OLs grows a little less than that of the wild-type strain at 37°C (Figure 5B). At 42°C, the *R. andropogonis* strains do not grow, apparently because they are lysed. No growth differences were observed between both *B. cenocepacia* strains at 30 and 37°C, but, at 42°C the absence of OL in *B. cenocepacia* NG1 apparently mildly affected the growth of this strain compared to the wild-type J2315 (Figure 5C).

With respect to acid stress, we observed that the presence of OL did not affect the growth of the *R. andropogonis* strains. In contrast, the absence of OL in *B. cenocepacia* NG1 mildly affected the stress tolerance of this strain in comparison with its respective wild-type J2315. Also, it was observed that *R. andropogonis* strains were more resistant to pH 4 than the strains of *B. cenocepacia* (Figures 5D,E).

Osmotic stress is a common environmental stress for *B. cenocepacia*, to which it is exposed for example in the lungs of patients with cystic fibrosis (CF) or in soil (Smith et al., 1996). Behrends et al. (2011), investigated the tolerance to osmotic stress of five isolates of *B. cenocepacia* and elucidated the metabolic changes associated with osmotic stress when the isolates were exposed to 0.5 M NaCl. When exposing the strains to NaCl concentrations of 0.05, 0.5, and 1 M, we observed that there was no difference between the strains that synthesized OLs with those that did not (Figures 5F–H). *R. andropogonis* did not grow under osmotic stress conditions, whereas *B. cenocepacia* strains grow well in medium supplemented with 0.5 M NaCl, but did not grow in medium supplemented with 1 M NaCl.

### The Absence of OLs in *B. cenocepacia* Reduces Their Virulence in a *Galleria mellonella* Infection Model Under Specific Conditions

**Figure 6:**
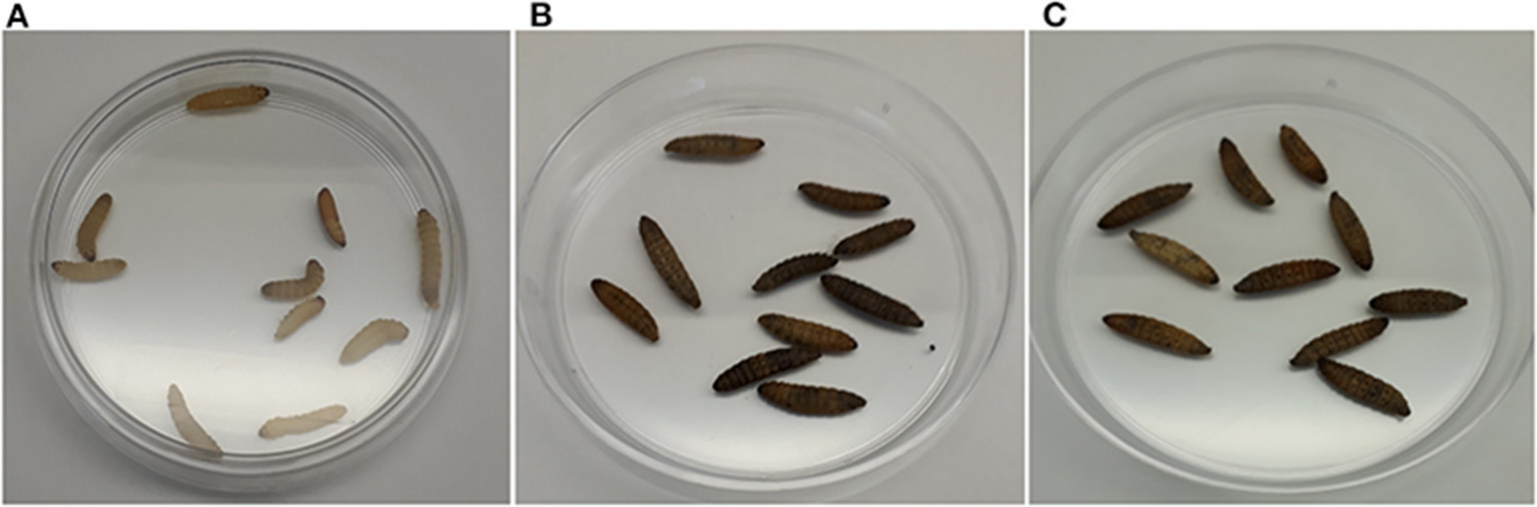
Melanization in *Galleria mellonella* larvae infected with strains of *B. cenocepacia* occurs in the presence of the pathogen and does not depend on the presence of OLs. **(A)** Control larvae infiltrated with 10 mM MgSO_4_, **(B)** Larvae infected with *B. cenocepacia* wild-type J2315, and **(C)** larvae infected with the *B. cenocepacia* mutant strain NG1. The pathogenicity assays were carried out using 10 insect larvae placed individually in 55 mm Petri dishes without diet and incubated at 30°C, evaluating mortality every 24 h after injection for 5 days before moving to the next larval growth stage. The melanization in *G. mellonella* larvae occurred along with larva death. Five independent experiments were performed.

**Figure 7:**
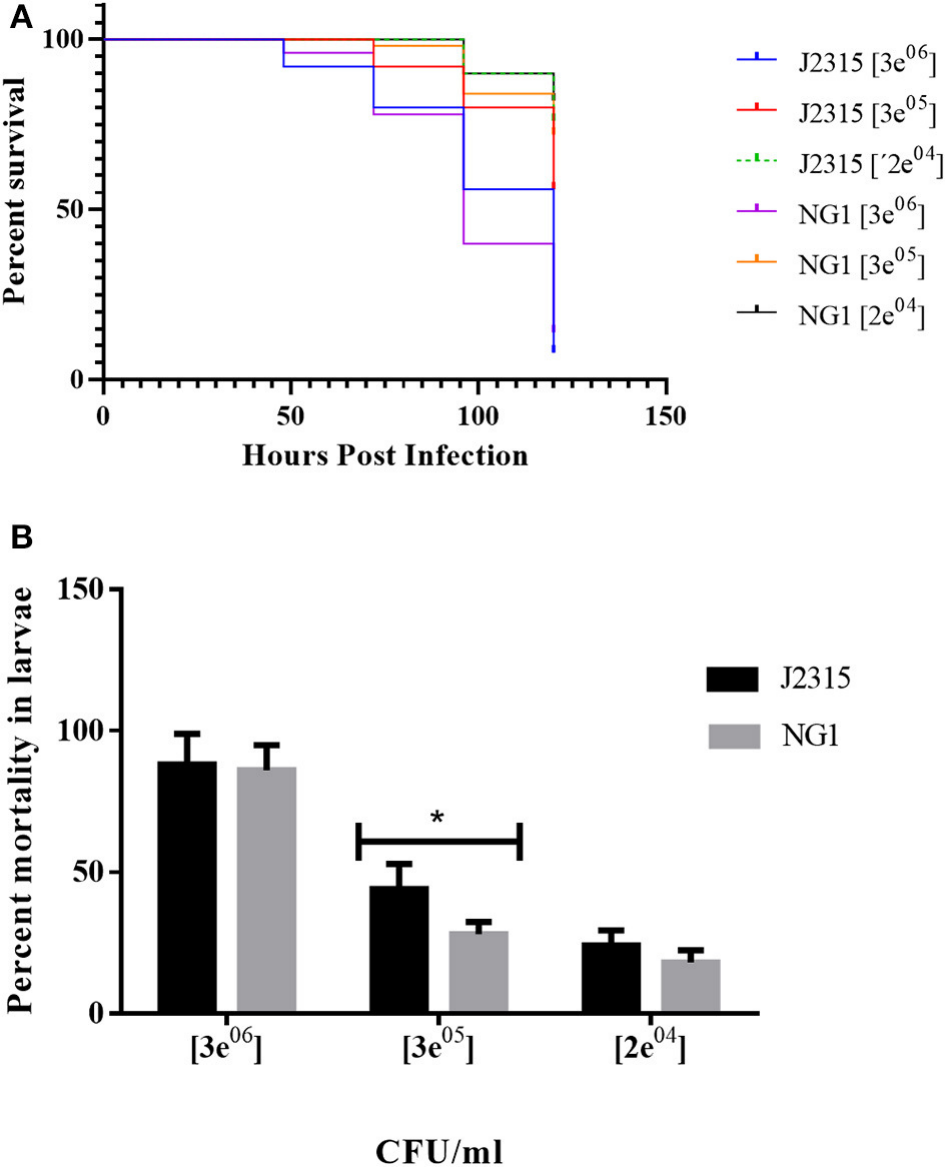
*B. cenocepacia* lacking OLs caused lower mortality in *Galleria mellonella* larvae. **(A)** Kaplan-Meier survival curves: Larvae were injected with wild-type cells of *B. cenocepacia* J2315 or the mutant strain NG1 that does not synthesize OLs at concentrations of 3 × 10^7^, 3 × 10^6^, 3 × 10^5^, and 2 × 10^4^ CFU/ml. Survival was evaluated every 24 h for 5 days. At the highest concentration of 3 × 10^7^ CFU/ml, the mortality for both strains was 100 %. **(B)** Strain J2315 (black columns) caused increased mortality after 5 days in the infection model of *G. mellonella* larvae compared with the mutant NG1 that lacking OLs (gray columns) at a concentration of 3 × 10^5^ CFU/ml and was significantly different (*) with an analysis tailed *t*-test with a *P*-value: 0.0349. The experiment was repeated five times with similar results using fifty larvae in total for each treatment.

Non-mammalian model systems of infection such as *G. mellonella* have been used to study the virulence of human pathogens such as *Candida albicans, Pseudomonas aeruginosa, Acinetobacter baumannii, Staphylococcus aureus, Enterococcus faecalis, Yersinia pseudotuberculosis*, and species of the genus *Burkholderia* (Fedhila et al., 2010). *G. mellonella* is a relatively cheap infection model, small in size, and possesses a short life cycle, the organisms can be handled easily and only small quantities of test compounds are required for injection. It has been used as a model for clinical infections as it can be maintained at physiological temperatures (37°C) for up to 5 days and its innate response to infections is structurally and functionally similar to that of mammals. The release of reactive oxygen species and antimicrobial peptides into the hemolymph is triggered by humoral responses. Hemolymph clotting, equivalent to mammalian blood clotting, is followed by melanization. The cellular response results in the encapsulation of the infecting pathogen and phagocytosis (McCloskey et al., 2019). When control larvae were infiltrated with saline solution, with *E. coli* or simply picketed, they were asymptomatic and neither death nor melanization were observed until day 5 (Figure 6A). When evaluating the virulence of the *B. cenocepacia* wild-type J2315 and the mutant NG1 in larvae of *G. mellonella*, both strains caused the typical melanization (Figures 6B,C). When larvae of *G. mellonella* were infected with high concentrations of 3 × 10^7^ CFU/ml and 3 × 10^6^ CFU/ml, no differences between the larvae infected with the wild-type or the mutant strain were observed (Figure 7). However, at lower bacterial concentrations such as 3 × 10^5^ CFU/ml, lower mortalities were observed in the case of the mutant lacking OLs. At this concentration, the wild-type strain *B. cenocepacia* J2315 caused a mortality rate of 44% after 5 days, while the mortality rate of the mutant strain NG1 was 28% after the same time. The minimum dose for larval mortality was 2 × 10^3^ CFU/ml and only those larvae infected with the wild-type strain (2% mortality) presented larval death. Our results indicate that the presence of OLs causes an increase in mortality in the *G. mellonella* infection model under a cell density of 3 × 10^5^ CFU/ml.

### The Presence of OLs Induces an Increased Accumulation of Reactive Oxygen Species (ROS)

**Figure 8:**
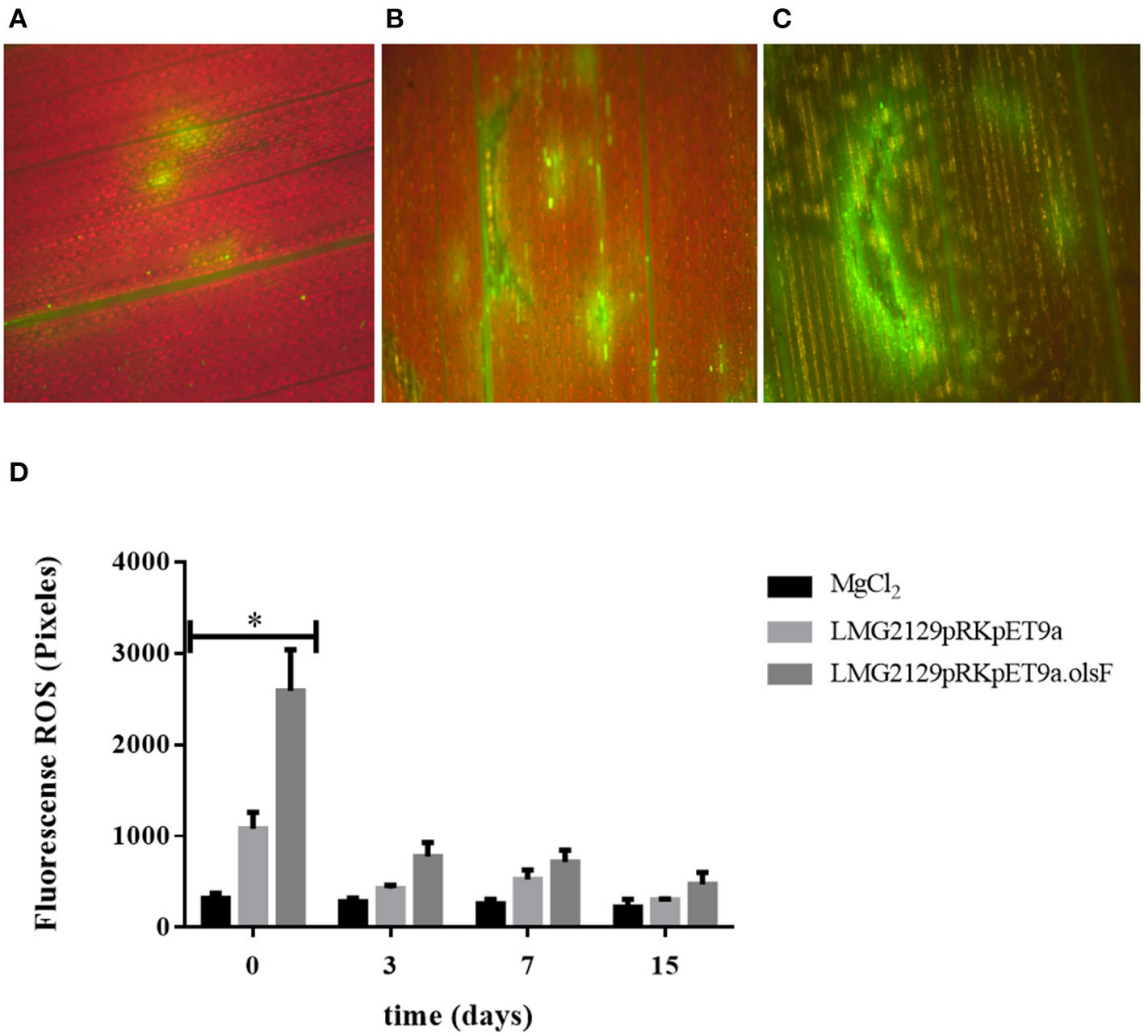
The presence of ornithine lipids in *R. andropogonis* drastically increases the formation of reactive oxygen species (ROS) directly after infiltration into leaves of maize plants. ROS detection as DCFH-DA fluorescence **(A)** in maize leaves treated with mock solution, **(B)** treated with LMG2129.pRK404.pET9a, or **(C)** treated with LMG2129.pRK404.pET9a.olsF. **(D)** Densitometric quantification of ROS production by measuring DCFH-DA fluorescence showed that immediately after infection (0 dpi) the ROS levels were two times higher in leaves infiltrated with the strain constitutively forming OLs compared to leaves infiltrated with the wild-type strain, and were significantly different (*) determined by the analysis tailed *t*-test with a *P*-value: 0,0263 at 0 dpi. ROS concentration was lower on later times at 3, 7, and 15 dpi, and no differences were detected between the treatments. The experiment was repeated three times with similar results.

Plant innate immunity is the first line of defense against multiple pathogens. An important part of these immune responses is the production of extracellular reactive oxygen species (ROS). ROS can be present as impermeable superoxide (${\text{O}}_{2}^{-}$) or as permeable hydrogen peroxide (H_2_O_2_) and it can be readily translocated from one cell to another (Ghosh et al., 2019). *B. andropogonis* LMG2129 has been described as bacterial stripe pathogen in maize and other plants (Moffett et al., 1986; Cother et al., 2004; Li and De Boer, 2005; Eloy and Cruz, 2008). We wanted to study if the presence or absence of OLs could modify the plant immune responses, in particular the ROS production by the plant upon infection with *R. andropogonis*. ROS formation in infiltrated plant leaves was quantified using the fluorescence emitted by the compound diacetate 2 ′, 7′-dichlorodihydrofluorescein (DCFH-DA) when it is oxidized by ROS (Lehmann et al., 2015). Directly after infection (0 days post infection (dpi) ROS formation is detected in leaves infected with *R. andropogonis* (Figure 8). Interestingly, ROS production was much higher in leaves infected with the strain LMG2129.pRK.pET9a.olsF constitutively forming OLs compared to mock-treated samples (MgCl_2_) and to the wild-type strain (Figures 8A–C). To study the progression of ROS accumulation over time, ROS was quantified at 0, 3, 7, and 15 dpi (Figure 8D). Immediately after infection (0 dpi), ROS levels were twice as high in the strain constitutively forming OLs than the wild-type strain. ROS levels decreased at 3, 7, and 15 dpi, and no significant differences were detected between the different treatments at the later time points. Wounding caused by infiltration has been also described to induce ROS accumulation (Benikhlef et al., 2013). We observed that the possible damage by infiltrating the mock solution only slightly induces ROS formation (Figures 8A,D). Our results indicate that the presence of OLs triggers a strong ROS accumulation.

### Constitutive OL Formation Does Not Modify the Progression of Infection Caused by *Robbsia andropogonis* on Its Host Maize

**Figure 9:**
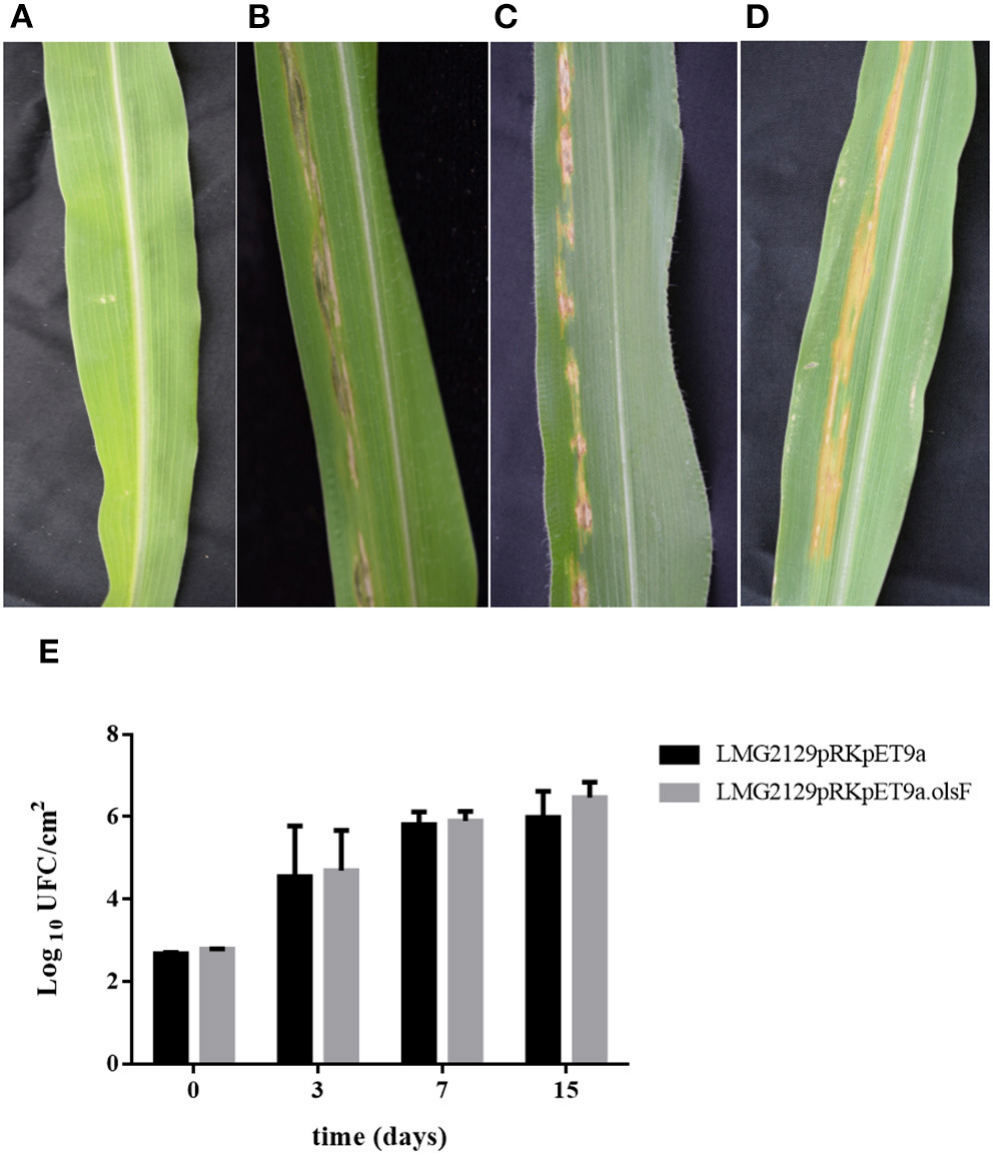
The presence of OLs does not modify the progression of the infection of *R. andropogonis* in maize leaves. When infiltrated with *R. andropogonis* strains, maize leaves showed dark-red lesions and chlorosis, while control plants infiltrated with the mock solution were asymptomatic. **(A)** Mock solution, **(B)** wild-type LMG2129, **(C)** LMG2129.pRK404.pET9a, and **(D)** LMG2129.pRK404.pET9a.olsF. **(E)** The number of bacteria isolated from the maize leaves at 0, 3, 7, and 15 dpi, increased over time in infected plants, and no significant differences could be observed between the strains. The experiment was repeated three times with similar results using thirty corn plants in total for each treatment.

Based on the increased accumulation of ROS caused by the OL-forming *R. andropogonis* strain on maize (Figure 8), we wanted to characterize if the presence of OLs would modify the progression of the infection of *R. andropogonis*. Disease symptoms (dark-red lesions and chlorosis) and the number of living bacteria present inside the leaves were evaluated (Figure 9). Maize leaves were inoculated with the *R. andropogonis* strains and 3 days post-inoculation (dpi) disease symptoms were observed at the point of inoculation. These lesions continued to advance along the veins during the following days, while control plants infiltrated with the mock solution were asymptomatic (Figure 9A). Remarkably, the lesions caused by strains constitutively expressing OlsF were similar to the ones produced by the wild-type strain *B. andropogonis* LMG2129 (Figures 9B–D). Bacteria were isolated from leaves and colony-forming units (CFU) were determined at 0, 3, 7, and 15 dpi. Bacterial numbers increased over time in infected plants and no significant differences could be observed between both strains (Figure 9E). These results suggest that the increased ROS accumulation induced by the overexpression of OLs did not influence the progression of the infection on maize.

## Discussion

The genus *Burkholderia* s.l. contains pathogenic, phytopathogenic, symbiotic and non-symbiotic species from a very wide range of environmental (soil, water, plants, fungi) and clinical (animal, human) habitats (Estrada-de Los Santos et al., 2018). One characteristic that was thought to be unique for the genus *Burkholderia* was the presence of the membrane lipids phosphatidylethanolamine and ornithine lipids (OLs), both in a hydroxylated and in a non-hydroxylated form (Yabuuchi et al., 1992). Here, we show that only *Burkholderia* s.s. species which often correspond to pathogens of humans, plants or animals constitutively form OLs and that these lipids are absent (or at least not constitutively formed) in the recently proposed genera *Paraburkholderia, Caballeronia, Robbsia, Trinickia*, and *Mycetohabitans* that are related to beneficial bacteria or that do not cause pathogenicity in humans. However, the absence of OLs is probably not caused by the absence of a copy of the *olsB* gene because we found that all analyzed species possessed a gene encoding a homolog of the *N*-acyltransferase OlsB (Bcal1281) responsible for the first step in OL synthesis in *B. cenocepacia* (González-Silva et al., 2011). The genomic context around the respective genes was usually conserved throughout *Burkholderia* s.l. (data not shown). The synthesis of OLs in the recently proposed genera is probably induced at low phosphate concentrations and regulated by PhoB as seen in a variety of other bacteria such as for example *Sinorhizobium meliloti, Rhodobacter sphaeroides, Serratia proteamaculans*, and *Vibrio cholerae*. This idea is supported by our prediction of Pho boxes preceding the respective putative *olsB* genes in 21 of the 43 genomes analyzed. The majority of these 21 genomes corresponded to genomes of bacteria that do not constitutively synthesize OLs (*Paraburkholderia* and *Caballeronia*), and five corresponded to bacterial species of the genus *Burkholderia* s.s. which constitutively synthesize OLs (Supplementary Table 4). It is possible that we did not detect the Pho boxes in some genomes, because of the evolutionary distance between α-proteobacteria and β-proteobacteria or alternatively that the genes responsible for OL formation are regulated differently in the other species. The presence of a Pho box in front of *olsB* in strains forming OLs constitutively might mean that expression of the respective *olsB* copies is increased further under phosphate-limiting conditions and that this leads to an increase in OL formation. Among the species that we identified with predicted Pho boxes was *P. xenovorans* and we cultured the strain LB400 (Goris et al., 2004) in minimal medium supplemented under low phosphate conditions. We observed that when cultivated in growth medium with 0 or 0.02 mM phosphate the strain was able to replace the phospholipids PE and 2OH-PE by modified and unmodified OLs (data not shown).

OLs can be modified by hydroxylations, either in the head group or in the fatty acid chains or by *N*-methylations (Sohlenkamp and Geiger, 2016). These modifications can play an important role in response to environmental stresses, and it has been observed that the amount of hydroxylated lipids (2OH-PE and 2OH-OL) increases at high temperatures in *B. cepacia* (Taylor et al., 1998) and that the hydroxylated OL NL1 is formed under acid stress in *B. cenocepacia* (González-Silva et al., 2011). In this study, we noticed that the absence of OLs in *B. cenocepacia* strain NG1, slightly affected tolerance to high temperature and acidic pH compared to its respective wild-type J2315 (Figures 5C,E) and we believe that the slight decrease in growth could be caused by the absence of the hydroxylated OL NL1. This observation is consistent with the earlier observations in *R. tropici*, where the presence of (hydroxylated) OLs conferred resistance to acid stress conditions and increased temperatures (Vences-Guzmán et al., 2011), although the effect observed in *B. cenocepacia* is clearly not as strong as in *R. tropici*. A possible explanation is that the presence/absence of OLs in *B. cenocepacia* is not a main factor for stress resistance as seems to be the case in *R. tropici*, but a secondary factor contributing on a smaller scale to resistance to the stress conditions studied. In the case of the *R. andropogonis* strains, the strain expressing OlsF constitutively is growing slower under all conditions and it does not contribute to an increased resistance to abiotic stress conditions under the chosen expression conditions.

Membrane lipids can play a role in bacteria-host relationships during pathogenicity. Bartholomew et al. (2019), described that a 2-hydroxylation in lipid A contributes to virulence in *Acinetobacter baumannii* using *G. mellonella* as an infection model. They could show that only 10 % of the larvae survived when challenged with the wild-type and the complemented mutant. In contrast, 50 % of the larvae survived when inoculated with the *lpxO* mutant. A recent study by Kim et al. (2018) concluded that an increased formation of OLs might play a role in increasing persistence, while at the same time reducing the virulence of *Pseudomonas aeruginosa* on *Tenebrio molitor* (an insect) and in *Caenorhabditis elegans* (nematode). In our study, we observed that the mean of the mortalities after 5 days caused by the *B. cenocepacia* mutant deficient in OL seems to be lower at different cell densities tested, but a statistically significant reduction of mortality in the *G. mellonella* model was only observed at a cell density of 3 × 10^5^ CFU/ml. A possible explanation is that OLs are not the main factor contributing to mortality in this experimental system, and that the effect of the absence of OLs might be covered by other unknown factors. Older studies by other groups about the immunogenicity of OLs have been contradictory and a probable reason is that OLs are not the only or main factor in all the bacteria studied, although they probably contribute to immunogenicity by interfering with or contributing to the effects of other virulence factors. We also observed that the resistance to a selected set of antibiotics and microbial peptides was not affected by the presence of OLs (data not shown). However, although OLs seem to affect the bacterial-host interaction, there are clearly additional factors present in *B. cenocepacia* affecting its virulence.

Plants have elaborate multilayered defense mechanisms to survive the constant attack of pathogens. The first line of defense is innate immunity, which is triggered by molecular components of microbes, called Microbe-Associated Molecular Patterns (MAMPs), including components of the bacterial membrane (Boller and Felix, 2009). Once MAMPs are recognized, the Pattern-Triggered Immunity (PTI) is activated, inducing a strong accumulation of ROS, MAPK signaling cascades and transcriptional activation of early defense response genes (Tsuda and Somssich, 2015). After this initial response, plants can induce the Effector-Triggered Immunity (ETI), which is based on the specific recognition of pathogen effectors by the *R* genes that leads to a local programmed cell death or hypersensitive response (HR) (Boller and Felix, 2009). The combined effect of PTI and ETI can block the invasion of pathogens, both locally at the infection site and systemically in uninfected leaves (Craig et al., 2009; Tsuda and Somssich, 2015). However, although PTI and ETI share similar molecular defense response elements, they have different dynamics in the activation and intensity (Katagiri and Tsuda, 2010). It was demonstrated that bacterial polar lipids can stimulate specific immune responses in the host (Melian et al., 2000; Roura-Mir et al., 2005). For instance, polar lipids of *B. pseudomallei* (e.g., ornithine lipids and rhamnolipids) induce antibody production and several polar lipids stimulate cellular immune responses (González-Juarrero et al., 2013). Here we show that *R. andropogonis* constitutively forming OLs causes an increased formation of ROS directly after infection of maize leaves (Figure 8), suggesting that plants can recognize OLs as MAMPs, inducing the PTI. As signaling molecules and as toxic compounds, ROS have been described to play multiple roles during plants life, including modulation of growth and development and response to abiotic and biotic stimuli (Camejo et al., 2016). The constitutive presence of OLs in *R. andropogonis* does not affect the number of bacteria growing inside the leaves (Figure 9), which suggests that a possible recognition of OLs and subsequent accumulation of ROS are not sufficient to stop the infection. One possibility to explain this phenomenon, is that the infection inflicted by *R. andropogonis* can be inhibited by activating the ETI but no PTI. This is in agreement with the observation that expression of the R gene Rxo1 controls the resistance against *R. andropogonis* in maize and rice (Zhao et al., 2005). Another possibility is that during the infection, *R. andropogonis* can produce scavenger molecules or detoxifying enzymes that can inhibit ROS, which has been previously described as invading strategy for multiple plant pathogens (Lehmann et al., 2015).

In conclusion, the presence of OLs in both bacteria-host interactions we studied affects how the pathogen is perceived and it may affect in some cases the outcome of the infection. Further work is required to determine the role of OLs in *Burkholderia* spp. during plant-pathogen and animal-pathogen interactions.

## Data Availability Statement

The raw data supporting the conclusions of this article will be made available by the authors, without undue reservation.

## Author Contributions

LAC-C, MS, and CS designed the study. LAC-C, RS-M, MT, LM-A, MÁV-G, and ZG carried out the experiments. LAC-C, LL, ZG, ED-G, MS, and CS carried out the data analysis and discussed the results. LAC-C and CS were involved in drafting the manuscript and all authors read and approved the final manuscript.

## Conflict of Interest

The authors declare that the research was conducted in the absence of any commercial or financial relationships that could be construed as a potential conflict of interest.

## References

[B1] Alghoribi M. F. Gibreel T. M. Dodgson A. R. Beatson S. A. Upton M. (2014). *Galleria mellonella* infection model demonstrates high lethality of ST69 and ST127 uropathogenic *E. coli*. PLoS ONE 9:e101547. 10.1371/journal.pone.0101547 25061819 PMC4111486

[B2] Barbosa L. C. Goulart C. L. Avellar M. M. Bisch P. M. von Kruger W. M. A. (2018). Accumulation of ornithine lipids in *Vibrio cholerae* under phosphate deprivation is dependent on VC0489 (OlsF) and PhoBR system. Microbiology 164, 395–399. 10.1099/mic.0.000607 29458678

[B3] Bartholomew T. L. Kidd T. J. Sa Pessoa J. Conde Alvarez R. Bengoechea J. A. (2019). 2-Hydroxylation of *Acinetobacter baumannii* Lipid A contributes to virulence. Infect. Immun. 87:e00066-19. 10.1128/IAI.00066-19 30745327 PMC6434125

[B4] Behrends V. Bundy J. G. Williams H. D. (2011). Differences in strategies to combat osmotic stress in *Burkholderia cenocepacia* elucidated by NMR-based metabolic profiling. Lett. Appl. Microbiol. 52, 619–625. 10.1111/j.1472-765X.2011.03050.x 21446999

[B5] Benikhlef L. L'Haridon F. Abou-Mansour E. Serrano M. Binda M. Costa A.. (2013). Perception of soft mechanical stress in *Arabidopsis* leaves activates disease resistance. BMC Plant Biol. 13:133. 10.1186/1471-2229-13-133 24033927 PMC3848705

[B6] Benning C. Huang Z. H. Gage D. A. (1995). Accumulation of a novel glycolipid and a betaine lipid in cells of *Rhodobacter sphaeroides* grown under phosphate limitation. Arch. Biochem. Biophys. 317, 103–111. 10.1006/abbi.1995.1141 7872771

[B7] Bligh E. G. Dyer W. J. (1959). A rapid method of total lipid extraction and purification. Can. J. Biochem. Physiol. 37, 911–917. 10.1139/o59-099 13671378

[B8] Boller T. Felix G. (2009). A renaissance of elicitors: perception of microbe-associated molecular patterns and danger signals by pattern-recognition receptors. Annu. Rev. Plant Biol. 60, 379–406. 10.1146/annurev.arplant.57.032905.105346 19400727

[B9] Bosak T. Schubotz F. de Santiago-Torio A. Kuehl J. V. Carlson H. K. Watson N.. (2016). System-wide adaptations of *Desulfovibrio alaskensis* G20 to phosphate-limited conditions. PLoS ONE 11:e0168719. 10.1371/journal.pone.0168719 28030630 PMC5193443

[B10] Camejo D. Guzman-Cedeno A. Moreno A. (2016). Reactive oxygen species, essential molecules, during plant-pathogen interactions. Plant Physiol. Biochem. 103, 10–23. 10.1016/j.plaphy.2016.02.035 26950921

[B11] Contreras-Moreira B. Vinuesa P. (2013). GET_HOMOLOGUES, a versatile software package for scalable and robust microbial pangenome analysis. Appl. Environ. Microbiol. 79, 7696–7701. 10.1128/AEM.02411-13 24096415 PMC3837814

[B12] Cother E. J. Noble D. Peters B. J. Albiston A. Ash G. J. (2004). A new bacterial disease of jojoba (*Simmondsia chinensis*) caused by *Burkholderia andropogonis*. Plant Pathol. 53, 129–135. 10.1111/j.0032-0862.2004.00982.x

[B13] Craig A. Ewan R. Mesmar J. Gudipati V. Sadanandom A. (2009). E3 ubiquitin ligases and plant innate immunity. J. Exp. Bot. 60, 1123–1132. 10.1093/jxb/erp059 19276192

[B14] Defrance M. van Helden J. (2009). Info-gibbs: a motif discovery algorithm that directly optimizes information content during sampling. Bioinformatics 25, 2715–2722. 10.1093/bioinformatics/btp490 19689955

[B15] Depoorter E. Bull M. J. Peeters C. Coenye T. Vandamme P. Mahenthiralingam E. (2016). *Burkholderia*: an update on taxonomy and biotechnological potential as antibiotic producers. Appl. Microbiol. Biotechnol. 100, 5215–5229. 10.1007/s00253-016-7520-x 27115756

[B16] Dobritsa A. P. Samadpour M. (2016). Transfer of eleven species of the genus Burkholderia to the genus Paraburkholderia and proposal of *Caballeronia* gen. nov. to accommodate twelve species of the genera *Burkholderia* and *Paraburkholderia*. Int. J. Syst. Evol. Microbiol. 66, 2836–2846. 10.1099/ijsem.0.001065 27054671

[B17] Eloy M. Cruz L. (2008). A new bacterial disease of carnation in Portugal caused by *Burkholderia andropogonis*. Rev. Ciências Agrárias 31, 89–95.

[B18] Elshafie H. S. Viggiani L. Mostafa M. S. El-Hashash M. A. Camele I. Bufo S. A. (2017). Biological activity and chemical identification of ornithine lipid produced by *Burkholderia gladioli* pv. agaricicola ICMP 11096 using LC-MS and NMR analyses. J. Biol. Res. Boll. Soc. Ital. Biol. Sperimentale 90, 96–103. 10.4081/jbr.2017.6534

[B19] Estrada-de Los Santos P. Palmer M. Chavez-Ramirez B. Beukes C. Steenkamp E. T. Briscoe L.. (2018). Whole genome analyses suggests that *Burkholderia* sensu lato contains two additional novel genera (*Mycetohabitans* gen. nov., and *Trinickia* gen. nov.): implications for the evolution of diazotrophy and nodulation in the *Burkholderiaceae*. Genes 9:389. 10.3390/genes9080389 30071618 PMC6116057

[B20] Fahraeus G. (1957). The infection of clover root hairs by nodule bacteria studied by a simple glass slide technique. J. Gen. Microbiol. 16, 374–381. 10.1099/00221287-16-2-374 13416514

[B21] Fedhila S. Buisson C. Dussurget O. Serror P. Glomski I. J. Liehl P.. (2010). Comparative analysis of the virulence of invertebrate and mammalian pathogenic bacteria in the oral insect infection model *Galleria mellonella*. J. Invertebr. Pathol. 103, 24–29. 10.1016/j.jip.2009.09.005 19800349

[B22] Gao J. L. Weissenmayer B. Taylor A. M. Thomas-Oates J. López-Lara I. M. Geiger O. (2004). Identification of a gene required for the formation of lyso-ornithine lipid, an intermediate in the biosynthesis of ornithine-containing lipids. Mol. Microbiol. 53, 1757–1770. 10.1111/j.1365-2958.2004.04240.x 15341653

[B23] Geiger O. González-Silva N. López-Lara I. M. Sohlenkamp C. (2010). Amino acid-containing membrane lipids in bacteria. Prog. Lipid Res. 49, 46–60. 10.1016/j.plipres.2009.08.002 19703488

[B24] Geiger O. Röhrs V. Weissenmayer B. Finan T. M. Thomas-Oates J. E. (1999). The regulator gene *phoB* mediates phosphate stress-controlled synthesis of the membrane lipid diacylglyceryl-*N,N,N*-trimethylhomoserine in *Rhizobium* (*Sinorhizobium*) *meliloti*. Mol. Microbiol. 32, 63–73. 10.1046/j.1365-2958.1999.01325.x 10216860

[B25] Geske T. Vom Dorp K. Dörmann P. Hölzl G. (2013). Accumulation of glycolipids and other non-phosphorous lipids in *Agrobacterium tumefaciens* grown under phosphate deprivation. Glycobiology 23, 69–80. 10.1093/glycob/cws124 22923441

[B26] Ghosh S. Malukani K. K. Chandan R. K. Sonti R. V. Jha G. (2019). How plants respond to pathogen attack: interaction and communication, in Sensory Biology of Plants, ed Sopory S. (Singapore: Springer), 537 10.1007/978-981-13-8922-1_20

[B27] Gillis M. Van T. V. Bardin R. Goor M. Hebbar P. Willems A. (1995). Polyphasic taxonomy in the genus *Burkholderia* leading to an emended description of the genus and proposition of *Burkholderia vietnamiensis* sp. nov. for N_2_-fixing isolates from rice in Vietnam. Int. J. Syst. Evol. Microbiol. 45, 274–289. 10.1099/00207713-45-2-274

[B28] González-Juarrero M. Mima N. Trunck L. A. Schweizer H. P. Bowen R. A. Dascher K.. (2013). Polar lipids of *Burkholderia pseudomallei* induce different host immune responses. PLoS ONE 8:e80368. 10.1371/journal.pone.0080368 24260378 PMC3832426

[B29] González-Silva N. López-Lara I. M. Reyes-Lamothe R. Taylor A. M. Sumpton D. Thomas-Oates J.. (2011). The dioxygenase-encoding *olsD* gene from *Burkholderia cenocepacia* causes the hydroxylation of the amide-linked fatty acyl moiety of ornithine-containing membrane lipids. Biochemistry 50, 6396–6408. 10.1021/bi200706v 21707055

[B30] Goris J. De Vos P. Caballero-Mellado J. Park J. Falsen E. Quensen J. F.. (2004). Classification of the biphenyl- and polychlorinated biphenyl-degrading strain LB400T and relatives as *Burkholderia xenovorans* sp. nov. Int. J. Syst. Evol. Microbiol. 54(Pt. 5), 1677–1681. 10.1099/ijs.0.63101-0 15388727

[B31] Guindon S. Dufayard J. F. Lefort V. Anisimova M. Hordijk W. Gascuel O. (2010). New algorithms and methods to estimate maximum-likelihood phylogenies: assessing the performance of PhyML 3.0. Syst. Biol. 59, 307–321. 10.1093/sysbio/syq010 20525638

[B32] Hanahan D. (1983). Studies on transformation of *Escherichia coli* with plasmids. J. Mol. Biol. 166, 557–580. 10.1016/S0022-2836(83)80284-8 6345791

[B33] Jonsson R. Struve C. Jenssen H. Krogfelt K. A. (2017). The wax moth *Galleria mellonella* as a novel model system to study Enteroaggregative *Escherichia coli* pathogenesis. Virulence 8, 1894–1899. 10.1080/21505594.2016.1256537 27824518 PMC5810504

[B34] Katagiri F. Thilmony R. He S. Y. (2002). The *Arabidopsis thaliana*-*Pseudomonas syringae* interaction. Arabidopsis Book 1:e0039. 10.1199/tab.0039 22303207 PMC3243347

[B35] Katagiri F. Tsuda K. (2010). Understanding the plant immune system. Mol. Plant Microbe Interact. 23, 1531–1536. 10.1094/MPMI-04-10-0099 20653410

[B36] Kim S. K. Park S. J. Li X. H. Choi Y. S. Im D. S. Lee J. H. (2018). Bacterial ornithine lipid, a surrogate membrane lipid under phosphate-limiting conditions, plays important roles in bacterial persistence and interaction with host. Environ. Microbiol. 20, 3992–4008. 10.1111/1462-2920.14430 30252196

[B37] Lehmann S. Serrano M. L'Haridon F. Tjamos S. E. Métraux J. P. (2015). Reactive oxygen species and plant resistance to fungal pathogens. Phytochemistry 112, 54–62. 10.1016/j.phytochem.2014.08.027 25264341

[B38] Lewenza S. Falsafi R. Bains M. Rohs P. Stupak J. Sprott G. D.. (2011). The *olsA* gene mediates the synthesis of an ornithine lipid in *Pseudomonas aeruginosa* during growth under phosphate-limiting conditions, but is not involved in antimicrobial peptide susceptibility. FEMS Microbiol. Lett. 320, 95–102. 10.1111/j.1574-6968.2011.02295.x 21535098

[B39] L'Haridon F. Besson-Bard A. Binda M. Serrano M. Abou-Mansour E. Balet F. (2011). A permeable cuticle is associated with the release of reactive oxygen species and induction of innate immunity. PLoS Pathog. 7:e1002148 10.1371/journal.ppat.1002148 21829351 PMC3145797

[B40] Li X. De Boer S. H. (2005). First report of *Burkholderia andropogonis* causing leaf spots of *Bougainvillea* sp. in Hong Kong and Clover in Canada. Plant Dis. 89:1132. 10.1094/PD-89-1132A 30791296

[B41] López-Lara I. M. Gao J. L. Soto M. J. Solares-Pérez A. Weissenmayer B. Sohlenkamp C.. (2005). Phosphorus-free membrane lipids of *Sinorhizobium meliloti* are not required for the symbiosis with alfalfa but contribute to increased cell yields under phosphorus-limiting conditions of growth. Mol. Plant Microbe Interact. 18, 973–982. 10.1094/MPMI-18-0973 16167767

[B42] Matus-Acuña V. Caballero-Flores G. Reyes-Hernández B. J. Martínez-Romero E. (2018). Bacterial preys and commensals condition the effects of bacteriovorus nematodes on *Zea mays* and *Arabidopsis thaliana*. Appl. Soil Ecol. 132, 99–106. 10.1016/j.apsoil.2018.08.012

[B43] McCloskey A. P. Lee M. Megaw J. McEvoy J. Coulter S. M. Pentlavalli S. (2019). Investigating the *in vivo* antimicrobial activity of a self-assembling peptide hydrogel using a *Galleria mellonella* infection model. ACS Omega 4, 99–106. 10.1021/acsomega.8b03578

[B44] Melian A. Watts G. F. Shamshiev A. De Libero G. Clatworthy A. Vincent M.. (2000). Molecular recognition of human CD1b antigen complexes: evidence for a common pattern of interaction with alpha beta TCRs. J. Immunol. 165, 4494–4504. 10.4049/jimmunol.165.8.4494 11035089

[B45] Miller J. (1972). Experiments in molecular genetics, in Experiments in Molecular Biology, eds Miller J. Miller J. B. (Cold Spring Harbor, NY: Cold Spring Harbor Laboratory Press), 431–433.

[B46] Minnikin D. E. Abdolrahimzadeh H. (1974). The replacement of phosphatidylethanolamine and acidic phospholipids by an ornithine-amide lipid and a minor phosphorus-free lipid in *Pseudomonas fluorescens* NCMB 129. FEBS Lett. 43, 257–260. 10.1016/0014-5793(74)80655-1 4213476

[B47] Moffett M. L. Hayward A. C. Fahy P. C. (1986). Five new hosts of *Pseudomonas andropogonis* occurring in eastern Australia: host range and characterization of isolates. Plant Pathol. 35, 34–43. 10.1111/j.1365-3059.1986.tb01978.x

[B48] Palleroni N. J. (2015). Burkholderia, in Bergey's Manual of Systematics of Archaea and Bacteria, eds Trujillo M. DeVos P. Hedlund B. Kämpfer P. Rainey F. Whitman W. B., 1–50. 10.1002/9781118960608

[B49] Rojas-Jiménez K. Sohlenkamp C. Geiger O. Martínez-Romero E. Werner D. Vinuesa P. (2005). A ClC chloride channel homolog and ornithine-containing membrane lipids of *Rhizobium tropici* CIAT899 are involved in symbiotic efficiency and acid tolerance. Mol. Plant Microbe Interact. 18, 1175–1185. 10.1094/MPMI-18-1175 16353552

[B50] Rojas-Rojas F. U. López-Sánchez D. Meza-Radilla G. Méndez-Canarios A. Ibarra J. A. Estrada-de Los Santos P. (2019). The controversial *Burkholderia cepacia* complex, a group of plant growth promoting species and plant, animals and human pathogens. Rev. Argent. Microbiol. 51, 84–92. 10.1016/j.ram.2018.01.002 29691107

[B51] Roura-Mir C. Wang L. Cheng T. Y. Matsunaga I. Dascher C. C. Peng S. L.. (2005). *Mycobacterium tuberculosis* regulates CD1 antigen presentation pathways through TLR-2. J. Immunol. 175, 1758–1766. 10.4049/jimmunol.175.3.1758 16034117

[B52] Sawana A. Adeolu M. Gupta R. S. (2014). Molecular signatures and phylogenomic analysis of the genus *Burkholderia*: proposal for division of this genus into the emended genus *Burkholderia* containing pathogenic organisms and a new genus *Paraburkholderia* gen. nov. harboring environmental species. Front. Genet. 5:429. 10.3389/fgene.2014.00429 25566316 PMC4271702

[B53] Simmons J. S. (1926). A culture medium for differentiating organisms of typhoid-colon aerogenes groups and for isolation of certain fungi: with colored plate. J. Infect. Dis. 39, 209–214. 10.1093/infdis/39.3.209

[B54] Simon R. Priefer U. Pühler A. (1983). A broad host range mobilization system for *in vivo* genetic engineering - transposon mutagenesis in gram-negative bacteria. Biotechnology 1, 784–791. 10.1038/nbt1183-784

[B55] Smith J. J. Travis S. M. Greenberg E. P. Welsh M. J. (1996). Cystic fibrosis airway epithelia fail to kill bacteria because of abnormal airway surface fluid. Cell 85, 229–236. 10.1016/S0092-8674(00)81099-5 8612275

[B56] Sohlenkamp C. Geiger O. (2016). Bacterial membrane lipids: diversity in structures and pathways. FEMS Microbiol. Rev. 40, 133–159. 10.1093/femsre/fuv008 25862689

[B57] Tahara Y. Fujiyoshi Y. (1994). A new method to measure bilayer thickness: cryo-electron microscopy of frozen hydrated liposomes and image simulation. Micron 25, 141–149. 10.1016/0968-4328(94)90039-6 8055245

[B58] Taylor C. J. Anderson A. J. Wilkinson S. G. (1998). Phenotypic variation of lipid composition in *Burkholderia cepacia*: a response to increased growth temperature is a greater content of 2-hydroxy acids in phosphatidylethanolamine and ornithine amide lipid. Microbiology 144(Pt. 7), 1737–1745. 10.1099/00221287-144-7-1737 9695908

[B59] Tsuda K. Somssich I. E. (2015). Transcriptional networks in plant immunity. New Phytol. 206, 932–947. 10.1111/nph.13286 25623163

[B60] Turatsinze J. V. Thomas-Chollier M. Defrance M. van Helden J. (2008). Using RSAT to scan genome sequences for transcription factor binding sites and cis-regulatory modules. Nat. Protoc. 3, 1578–1588. 10.1038/nprot.2008.97 18802439

[B61] Vandamme P. Holmes B. Coenye T. Goris J. Mahenthiralingam E. LiPuma J. J.. (2003). *Burkholderia cenocepacia* sp. nov.–a new twist to an old story. Res. Microbiol. 154, 91–96. 10.1016/S0923-2508(03)00026-3 12648723

[B62] Vences-Guzmán M. A. Guan Z. Escobedo-Hinojosa W. I. Bermúdez-Barrientos J. R. Geiger O. Sohlenkamp C. (2015). Discovery of a bifunctional acyltransferase responsible for ornithine lipid synthesis in *Serratia proteamaculans*. Environ. Microbiol. 17, 1487–1496. 10.1111/1462-2920.12562 25040623

[B63] Vences-Guzmán M. A. Guan Z. Ormeno-Orrillo E. González-Silva N. López-Lara I. M. Martínez-Romero E.. (2011). Hydroxylated ornithine lipids increase stress tolerance in *Rhizobium tropici* CIAT899. Mol. Microbiol. 79, 1496–1514. 10.1111/j.1365-2958.2011.07535.x 21205018 PMC3053409

[B64] Vinuesa P. Ochoa-Sánchez L. E. Contreras-Moreira B. (2018). GET_PHYLOMARKERS, a Software package to select optimal orthologous clusters for phylogenomics and inferring pan-genome phylogenies, used for a critical geno-taxonomic revision of the genus *Stenotrophomonas*. Front. Microbiol. 9:771. 10.3389/fmicb.2018.00771 29765358 PMC5938378

[B65] Weissenmayer B. Gao J. L. López-Lara I. M. Geiger O. (2002). Identification of a gene required for the biosynthesis of ornithine-derived lipids. Mol. Microbiol. 45, 721–733. 10.1046/j.1365-2958.2002.03043.x 12139618

[B66] Yabuuchi E. Kosako Y. Oyaizu H. Yano I. Hotta H. Hashimoto Y.. (1992). Proposal of *Burkholderia* gen. nov. and transfer of seven species of the genus *Pseudomonas* homology group II to the new genus, with the type species *Burkholderia cepacia* (Palleroni and Holmes 1981) comb. nov. Microbiol. Immunol. 36, 1251–1275. 10.1111/j.1348-0421.1992.tb02129.x 1283774

[B67] Yuan Z. C. Zaheer R. Morton R. Finan T. M. (2006). Genome prediction of PhoB regulated promoters in *Sinorhizobium meliloti* and twelve proteobacteria. Nucleic Acids Res. 34, 2686–2697. 10.1093/nar/gkl365 16717279 PMC1464414

[B68] Zhao B. Lin X. Poland J. Trick H. Leach J. Hulbert S. (2005). A maize resistance gene functions against bacterial streak disease in rice. Proc. Natl. Acad. Sci. U.S.A. 102, 15383–15388. 10.1073/pnas.0503023102 16230639 PMC1266081

