# Supplementary material for: Ornithine Lipids in Burkholderia spp. Pathogenicity
Source: Front Mol Biosci. 2021 Jan 5;7:610932. doi: 10.3389/fmolb.2020.610932 (PMC7814305; doi:10.3389/fmolb.2020.610932)
Supplement: Supplementary file 1 [file Table_1.DOCX]

**ORNITHINE LIPIDS IN *Burkholderia* spp. PATHOGENICITY**

***SUPPLEMENTARY MATERIAL***

**Table S1. Bacterial strains and plasmids used in this study.**

| **Strain or plasmid** | **Description** | **Reference** |
| --- | --- | --- |
| *Strains* |  |  |
| *E. coli* DH5α | strain used as a negative control in virulence assays with larvae of *G. mellonella* | (Hanahan, 1983) |
| *E. coli* S17-1 | strain used for conjugative plasmid transfer | (Simon et al., 1983) |
| *B. cenocepacia* NG1 | mutant *olsB*::*cat* | (González-Silva et al., 2011) |
| *B. cenocepacia* J2315 | wild-type | (Vandamme et al., 2003) |
| *R. andropogonis* LMG2129 | wild-type | (Gillis et al., 1995) |
| *B. cepacia* LMG25416 | wild-type | (Palleroni and Holmes, 1981) |
| *B. vietnamensis* TVV75 | wild-type | (Govindarajan et al., 2006) |
| *P. unamae* MTI641 | wild-type | (Caballero-Mellado et al., 2004) |
| *P. silvatlantica* SRMrh-20 | wild-type | (Perin et al., 2006) |
| *P. kururiensis* Kp23 | wild-type | (Estrada-De Los Santos et al., 2001) |
| *P. tropica* Ppe8 | wild-type | (Reis et al., 2004) |
| *P. ferrariae* FeG101 | wild-type | (Valverde et al., 2006) |
| *C. sordidicola* LMG22029 | wild-type | (Lim et al., 2003) |
| *P. phymatum* STM815 | wild-type | (Vandamme et al., 2002) |
| *P. tuberum* STM678 | wild-type | (Vandamme et al., 2002) |
| *P. nodosa* Br3437 | wild-type | (Chen et al., 2005; Chen et al., 2007) |
| *P. sacchari* LMG19450 | wild-type | (Brämer et al., 2001) |
| *P. graminis* C4D1MT | wild-type | (Viallard et al., 1998) |
| *P. caribensis* MWAP64T | wild-type | (Achouak et al., 1999) |
| *P. phytofirmans* PsJN | wild-type | (Barka et al., 2006) |
| *C. glathei* LMG14190 | wild-type | (Vandamme et al., 1997) |
| *P. fungorum* LMG16225 | wild-type | (Coenye et al., 2001) |
| *P. caledonica* LMG19076 | wild-type | (Coenye et al., 2001) |
| *P. hospita* LMG20598 | wild-type | (Goris et al., 2002) |
| *B. stabilis* LMG14294 | wild-type | (Vandamme et al., 2000) |
| *B. dolosa* LMG18943 | wild-type | (Vermis et al., 2004) |
| *B. gladioli* LMG2216 | wild-type | (Clode et al., 1999) |
| *P. ginsengisoli* LMG24044 | wild-type | (Kim et al., 2006) |
| *B. multivorans* LMG13010 | wild-type | (Vandamme et al., 1997) |
| *P. briophila* LMG23644 | wild-type | (Vandamme et al., 2007) |
| *B. latens* LMG24064 | wild-type | (Vanlaere et al., 2008a) |
| *P. megapolitana* LMG23650 | wild-type | (Vandamme et al., 2007) |
| *P. sartisoli* LMG24000 | wild-type | (Vanlaere et al., 2008b) |
| *B. seminalis* LMG24067 | wild-type | (Vanlaere et al., 2008a) |
| *T. caryophylli* LMG2155 | wild-type | (Glagoleva et al., 1996) |
| *B. plantarii* LMG9035 | wild-type | (Azegami et al., 1987; Urakami et al., 1994) |
| *Plasmids* |  |  |
| pRK404.pET9a | empty vector, broad host range plasmid pRK404 fused with expression vector pET9a | This work |
| pRK404.pET9a.olsF | vector with the *Serratia* *proteamaculans* *olsF* gene | This work |

**Table S2. Genomes** of ***Burkholderia* s.l. species downloaded from the NCBI RefSeq database.**

| **RefSeq** | **Organism** | **Strain** |
| --- | --- | --- |
| GCF_000009485.1 | *Burkholderia cenocepacia* | J2315 |
| GCF_000010545.1 | *Burkholderia multivorans* | ATCC 17616 |
| GCF_000011545.1 | *Burkholderia pseudomallei* | K96243 |
| GCF_000011705.1 | *Burkholderia mallei* | ATCC 23344 |
| GCF_000012945.1 | *Burkholderia lata* | 383 |
| GCF_000013645.1 | *Paraburkholderia xenovorans* | LB400 |
| GCF_000020045.1 | *Paraburkholderia phymatum* | STM815 |
| GCF_000020125.1 | *Paraburkholderia phytofirmans* | PsJN |
| GCF_000022645.2 | *Burkholderia glumae* | BGR1 |
| GCF_000152585.1 | *Burkholderia dolosa* | AU0158 |
| GCF_000172415.1 | *Paraburkholderia graminis* | C4D1M |
| GCF_000203915.1 | *Burkholderia ambifaria* | AMMD |
| GCF_000519185.1 | *Paraburkholderia nodosa* | DSM 21604 |
| GCF_000648925.1 | *Caballeronia jiangsuensis* | MP-1 |
| GCF_000685035.1 | *Paraburkholderia ferrariae* | NBRC 106233 |
| GCF_000685055.1 | *Paraburkholderia fungorum* | NBRC 102489 |
| GCF_000698555.1 | *Caballeronia grimmiae* | R27 |
| GCF_000698575.1 | *Caballeronia zhejiangensis* | OP- 1 |
| GCF_000698595.1 | *Caballeronia glathei* | DSM 50014 |
| GCF_000739735.1 | *Paraburkholderia ginsengisoli* | NBRC 100965 |
| GCF_000739795.1 | *Paraburkholderia kururiensis* subsp. *thiooxydans* | NBRC 107107 |
| GCF_000785435.1 | *Paraburkholderia sacchari* | LMG 19450 |
| GCF_000835205.1 | *Burkholderia plantarii* | PG1 |
| GCF_000959245.1 | *Burkholderia ubonensis* | MSMB22 |
| GCF_000959445.1 | *Burkholderia vietnamiensis* | LMG 10929 |
| GCF_000959725.1 | *Burkholderia gladioli* | ATCC 10248 |
| GCF_000970345.1 | *Robbsia andropogonis* | ICMP2807 |
| GCF_001278535.1 | *Caballeronia cordobensis* | LMG 27620 |
| GCF_001411495.1 | *Burkholderia cepacia* | ATCC 25416 |
| GCF_001449005.1 | *Paraburkholderia caribensis* | MWAP64 |
| GCF_001524085.1 | *Burkholderia seminalis* | FL-5-5-10-S1-D0 |
| GCF_001544455.2 | *Caballeronia sordidicola* | LMG 22029 |
| GCF_001544475.1 | *Caballeronia humi* | LMG 22934 |
| GCF_001544535.1 | *Caballeronia choica* | LMG 22940 |
| GCF_001544555.2 | *Caballeronia udeis* | LMG 27134 |
| GCF_001685505.1 | *Burkholderia stabilis* | LA20W |
| GCF_002071575.1 | *Paraburkholderia tropica* | Ppe8 |
| GCF_003096875.1 | *Paraburkholderia unamae* | SCZa-39 |
| GCF_003330745.1 | *Paraburkholderia caledonica* | PHRS4 |
| GCF_900101795.1 | *Paraburkholderia tuberum* | DUS833 |
| GCF_900107685.1 | *Paraburkholderia sartisoli* | LMG 24000 |
| GCF_900113825.1 | *Paraburkholderia megapolitana* | LMG 23650 |
| GCF_900177465.1 | *Paraburkholderia caryophylli* | Ballard 720 |

**Table S3.** **Sequence of Pho box motifs of the promoter regions of *Sinorhizobium* *meliloti*, *Mesorhizobium* *loti* and *Agrobacterium* *tumefaciens* genes used to construct the position weight matrix (Figure 3A) to search the *Burkholderia* s.l. genomes.**

| Gene | Pho box motif | Genome source | Reference |
| --- | --- | --- | --- |
| SMc01848 (BtaA) | TCGTCATCAAAGTGTAGC | *S. meliloti* | (Yuan et al., 2006) |
| mlr1574 (BtaA-like) | CTGTCACCGGCCTGTCAT | *M. loti* | (Yuan et al., 2006) |
| Atu1808 (Pgt) | ATGACATGTAACTGTCAC | *A. tumefaciens* | (Geske et al., 2013) |
| Atu0318 (OlsE) | CAGTCTTCAACCGGTCGC | *A. tumefaciens* | (Geske et al., 2013) |
| Atu2119 (BtaA) | CTGTCATCAAACTGTAGC | *A. tumefaciens* | (Geske et al., 2013) |

**Table S4**. **Putative Pho boxes identified in the promoter region upstream of genes encoding OlsB homologues in *Burkholderia* s.l. strains.**

| ***Species*** | **Start** | **end** | **sequence** | **weight** | **P val ▾** |
| --- | --- | --- | --- | --- | --- |
| *P. xenovorans* | -99 | -82 | CAGTCATCCAATCGTCAT | 7.5 | 5.2e-05 |
| *P. phytofirmans* | -100 | -83 | CAGTCATCCAATCGTCAT | 7.5 | 5.2e-05 |
| *P. fungorum* | -99 | -82 | CCGTCACCTAATCGTCAT | 7.5 | 5.2e-05 |
| *P. caledonia* | -99 | -82 | CAGTCATCCAATCGTCAT | 7.5 | 5.2e-05 |
| *B. multivorans* | -99 | -82 | ACGTCATTGAACGGACAC | 7.5 | 5.2e-05 |
| *P. kururiensis* | -77 | -60 | TTGCCATGGAAGCGTCAC | 7.9 | 3.7e-05 |
| *P. sartisoli* | -52 | -35 | ACGTCATCGAATCGTCAT | 8.2 | 2.8e-05 |
| *P. nodosa* | -95 | -78 | ACGTCATCGAATCGTCAT | 8.2 | 2.8e-05 |
| *P. tuberum* | -100 | -83 | CAGTCATCCAATCGTCAC | 8.4 | 2.4e-05 |
| *P. graminis* | -175 | -158 | CAGTCATCCAATCGTCAC | 8.4 | 2.4e-05 |
| *B. plantarii* | -99 | -82 | ATGTCACCGAATTGACAC | 8.6 | 2.0e-05 |
| *B. glumae* | -99 | -82 | ATGTCACCGAATTGACAC | 8.6 | 2.0e-05 |
| *C. humi* | -94 | -77 | ATTTCATCGAAGCGTCAC | 8.9 | 1.5e-05 |
| *C. cordobensis* | -112 | -95 | CCGTCATGCGCGTGTCGT | 8.9 | 1.5e-05 |
| *P. unamae* | -75 | -58 | TTGCCATGTAACCGTAAC | 9.0 | 1.4e-05 |
| *P. ferrariae* | -75 | -58 | ATGCCATGTAACCGTAAC | 9.0 | 1.4e-05 |
| *P. phymatum* | -99 | -82 | ACGTCATCGAATCGTCAC | 9.1 | 1.2e-05 |
| *P. kururiensis* | -99 | -82 | ACGTCATCGAATCGTCAC | 9.1 | 1.2e-05 |
| *P. caribensis* | -98 | -81 | ACGTCATCGAATCGTCAC | 9.1 | 1.2e-05 |
| *B. ubonensis* | -94 | -77 | TTGCCACGTAACGGTCAC | 9.2 | 1.1e-05 |
| *B. gladioli* | -99 | -82 | ATGTCACCAAACTGACAC | 12.4 | 3.7e-07 |

**References for supplementary material**

Achouak, W., Christen, R., Barakat, M., Martel, M.H., and Heulin, T. (1999). *Burkholderia caribensis* sp. nov., an exopolysaccharide-producing bacterium isolated from vertisol microaggregates in Martinique. *Int J Syst Bacteriol* 49 Pt 2**,** 787-794. doi: 10.1099/00207713-49-2-787.

Azegami, K., Nishiyama, K., Watanabe, Y., Kadota, I., Ohuchi, A., and Fukazawa, C. (1987). *Pseudomonas plantarii* sp. nov., the Causal Agent of Rice Seedling Blight *International Journal of Systematic and Evolutionary Microbiology* 37(2)**,** 144-152. doi: <https://doi.org/10.1099/00207713-37-2-144>.

Barka, E.A., Nowak, J., and Clément, C. (2006). Enhancement of Chilling Resistance of Inoculated Grapevine Plantlets with a Plant Growth-Promoting Rhizobacterium, *Burkholderia phytofirmans* Strain PsJN. *Applied and Environmental Microbiology* 72(11)**,** 7246-7252. doi: DOI: 10.1128/AEM.01047-06.

Brämer, C.O., Vandamme, P., da Silva, L.F., Gómez, J.G., and Steinbüchel, A. (2001). Polyhydroxyalkanoate-accumulating bacterium isolated from soil of a sugar-cane plantation in Brazil. *Int J Syst Evol Microbiol* 51(Pt 5)**,** 1709-1713. doi: 10.1099/00207713-51-5-1709.

Caballero-Mellado, J., Martinez-Aguilar, L., Paredes-Valdez, G., and Santos, P.E.L. (2004). *Burkholderia unamae* sp. nov., an N2-fixing rhizospheric and endophytic species. *Int J Syst Evol Microbiol* 54(Pt 4)**,** 1165-1172. doi: 10.1099/ijs.0.02951-0.

Clode, F.E., Metherell, L.A., and Pitt, T.L. (1999). Nosocomial Acquisition of *Burkholderia gladioli* in patients with cystic fibrosis. *Am J Respir Crit Care Med* 160(1)**,** 374-375. doi: 10.1164/ajrccm.160.1.16011.

Coenye, T., Laevens, S., Willems, A., Ohlen, M., Hannant, W., Govan, J.R., et al. (2001). *Burkholderia fungorum* sp. nov. and *Burkholderia caledonica* sp. nov., two new species isolated from the environment, animals and human clinical samples. *Int J Syst Evol Microbiol* 51(Pt 3)**,** 1099-1107. doi: 10.1099/00207713-51-3-1099.

Chen, W.M., de Faria, S.M., James, E.K., Elliott, G.N., Lin, K.Y., Chou, J.H., et al. (2007). *Burkholderia nodosa* sp. nov., isolated from root nodules of the woody Brazilian legumes *Mimosa bimucronata* and *Mimosa scabrella*. *Int J Syst Evol Microbiol* 57(Pt 5)**,** 1055-1059. doi: 10.1099/ijs.0.64873-0.

Chen, W.M., James, E.K., Chou, J.H., Sheu, S.Y., Yang, S.Z., and Sprent, J.I. (2005). Beta-rhizobia from *Mimosa pigra*, a newly discovered invasive plant in Taiwan. *New Phytol* 168(3)**,** 661-675. doi: 10.1111/j.1469-8137.2005.01533.x.

Estrada-De Los Santos, P., Bustillos-Cristales, R., and Caballero-Mellado, J. (2001). *Burkholderia*, a genus rich in plant-associated nitrogen fixers with wide environmental and geographic distribution. *Appl Environ Microbiol* 67(6)**,** 2790-2798. doi: 10.1128/AEM.67.6.2790-2798.2001.

Geske, T., Vom Dorp, K., Dörmann, P., and Hölzl, G. (2013). Accumulation of glycolipids and other non-phosphorous lipids in *Agrobacterium tumefaciens* grown under phosphate deprivation. *Glycobiology* 23(1)**,** 69-80. doi: 10.1093/glycob/cws124.

Gillis, M., Van, T.V., Bardin, R., Goor, M., Hebbar, P., Willems, A., et al. (1995). Polyphasic Taxonomy in the Genus *Burkholderia* Leading to an Emended Description of the Genus and Proposition of *Burkholderia vietnamiensis* sp. nov. for N_2_-Fixing Isolates from Rice in Vietnam. *International Journal of Systematic and Evolutionary Microbiology* 45(2)**,** 274-289. doi: doi.org/10.1099/00207713-45-2-274.

Glagoleva, O.B., Kovalskaya, N.U., and Umarov, M.M. (1996). Endosymbiosis formation between nitrogen-fixing bacteria pseudomonas caryophylli and rape root cells. *Endocytobiosis and Cell Research* 11**,** 147-158.

González-Silva, N., López-Lara, I.M., Reyes-Lamothe, R., Taylor, A.M., Sumpton, D., Thomas-Oates, J., et al. (2011). The dioxygenase-encoding *olsD* gene from *Burkholderia cenocepacia* causes the hydroxylation of the amide-linked fatty acyl moiety of ornithine-containing membrane lipids. *Biochemistry* 50(29)**,** 6396-6408. doi: 10.1021/bi200706v.

Goris, J., Dejonghe, W., Falsen, E., De Clerck, E., Geeraerts, B., Willems, A., et al. (2002). Diversity of transconjugants that acquired plasmid pJP4 or pEMT1 after inoculation of a donor strain in the A- and B-horizon of an agricultural soil and description of *Burkholderia hospita* sp. nov. and *Burkholderia terricola* sp. nov. *Syst Appl Microbiol* 25(3)**,** 340-352. doi: 10.1078/0723-2020-00134.

Govindarajan, M., Balandreau, J., Muthukumarasamy, R., Revathi, G., and Lakshminarasimhan, C. (2006). Improved Yield of Micropropagated Sugarcane Following Inoculation by Endophytic *Burkholderia vietnamiensis*. *Plant and Soil* 280**,** 239-252. doi: doi.org/10.1007/s11104-005-3223-2.

Hanahan, D. (1983). Studies on transformation of *Escherichia coli* with plasmids. *J Mol Biol* 166(4)**,** 557-580.

Kim, H.B., Park, M.J., Yang, H.C., An, D.S., Jin, H.Z., and Yang, D.C. (2006). *Burkholderia ginsengisoli* sp. nov., a beta-glucosidase-producing bacterium isolated from soil of a ginseng field. *Int J Syst Evol Microbiol* 56(Pt 11)**,** 2529-2533. doi: 10.1099/ijs.0.64387-0.

Lim, Y.W., Baik, K.S., Han, S.K., Kim, S.B., and Bae, K.S. (2003). *Burkholderia sordidicola* sp. nov., isolated from the white-rot fungus *Phanerochaete sordida*. *Int J Syst Evol Microbiol* 53(Pt 5)**,** 1631-1636. doi: 10.1099/ijs.0.02456-0.

Palleroni, N.J., and Holmes, B. (1981). Pseudomonas cepacia sp. nov., nom. rev. *International Journal of Systematic Bacteriology* 31(4)**,** 479-481. doi: doi.org/10.1099/00207713-31-4-479.

Perin, L., Martinez-Aguilar, L., Paredes-Valdez, G., Baldani, J.I., Estrada-de Los Santos, P., Reis, V.M., et al. (2006). *Burkholderia silvatlantica* sp. nov., a diazotrophic bacterium associated with sugar cane and maize. *Int J Syst Evol Microbiol* 56(Pt 8)**,** 1931-1937. doi: 10.1099/ijs.0.64362-0.

Reis, V.M., Santos, P.E.L., Tenorio-Salgado, S., Vogel, J., Stoffels, M., Guyon, S., et al. (2004). *Burkholderia tropica* sp. nov., a novel nitrogen-fixing, plant-associated bacterium. *Int J Syst Evol Microbiol* 54(Pt 6)**,** 2155-2162. doi: 10.1099/ijs.0.02879-0.

Simon, R., Priefer, U., and Pühler, A. (1983). A Broad Host Range Mobilization System for in vivo Genetic Engineering - Transposon Mutagenesis in Gram-Negative Bacteria. *Bio-Technology* 1(9)**,** 784-791. doi: DOI 10.1038/nbt1183-784.

Urakami, T., Ito-Yoshida, C., Araki, H., Kijima, T., Suzuki, K.-I., and Komagata, K. (1994). Transfer of *Pseudomonas plantarii* and *Pseudomonas glumae* to *Burkholderia* as *Burkholderia* spp. and Description of *Burkholderia vandii* sp. nov. *International Journal of Systematic and Evolutionary Microbiology* 44(2)**,** 235-245. doi: doi.org/10.1099/00207713-44-2-235.

Valverde, A., Delvasto, P., Peix, A., Velazquez, E., Santa-Regina, I., Ballester, A., et al. (2006). *Burkholderia ferrariae* sp. nov., isolated from an iron ore in Brazil. *Int J Syst Evol Microbiol* 56(Pt 10)**,** 2421-2425. doi: 10.1099/ijs.0.64498-0.

Vandamme, P., Goris, J., Chen, W.M., de Vos, P., and Willems, A. (2002). *Burkholderia tuberum* sp. nov. and *Burkholderia phymatum* sp. nov., nodulate the roots of tropical legumes. *Syst Appl Microbiol* 25(4)**,** 507-512. doi: 10.1078/07232020260517634.

Vandamme, P., Holmes, B., Coenye, T., Goris, J., Mahenthiralingam, E., LiPuma, J.J., et al. (2003). *Burkholderia cenocepacia* sp. nov.--a new twist to an old story. *Res Microbiol* 154(2)**,** 91-96. doi: 10.1016/S0923-2508(03)00026-3.

Vandamme, P., Holmes, B., Vancanneyt, M., Coenye, T., Hoste, B., Coopman, R., et al. (1997). Occurrence of multiple genomovars of *Burkholderia cepacia* in cystic fibrosis patients and proposal of *Burkholderia multivorans* sp. nov. *Int J Syst Bacteriol* 47(4)**,** 1188-1200. doi: 10.1099/00207713-47-4-1188.

Vandamme, P., Mahenthiralingam, E., Holmes, B., Coenye, T., Hoste, B., De Vos, P., et al. (2000). Identification and population structure of *Burkholderia stabilis* sp. nov. (formerly *Burkholderia cepacia* genomovar IV). *J Clin Microbiol* 38(3)**,** 1042-1047. doi: 10.1128/JCM.38.3.1042-1047.2000.

Vandamme, P., Opelt, K., Knochel, N., Berg, C., Schonmann, S., De Brandt, E., et al. (2007). *Burkholderia bryophila* sp. nov. and *Burkholderia megapolitana* sp. nov., moss-associated species with antifungal and plant-growth-promoting properties. *Int J Syst Evol Microbiol* 57(Pt 10)**,** 2228-2235. doi: 10.1099/ijs.0.65142-0.

Vanlaere, E., Lipuma, J.J., Baldwin, A., Henry, D., De Brandt, E., Mahenthiralingam, E., et al. (2008a). *Burkholderia latens* sp. nov., *Burkholderia diffusa* sp. nov., *Burkholderia arboris* sp. nov., *Burkholderia seminalis* sp. nov. and *Burkholderia metallica* sp. nov., novel species within the *Burkholderia cepacia* complex. *Int J Syst Evol Microbiol* 58(Pt 7)**,** 1580-1590. doi: 10.1099/ijs.0.65634-0.

Vanlaere, E., van der Meer, J.R., Falsen, E., Salles, J.F., de Brandt, E., and Vandamme, P. (2008b). *Burkholderia sartisoli* sp. nov., isolated from a polycyclic aromatic hydrocarbon-contaminated soil. *Int J Syst Evol Microbiol* 58(Pt 2)**,** 420-423. doi: 10.1099/ijs.0.65451-0.

Vermis, K., Coenye, T., LiPuma, J.J., Mahenthiralingam, E., Nelis, H.J., and Vandamme, P. (2004). Proposal to accommodate *Burkholderia cepacia* genomovar VI as *Burkholderia dolosa* sp. nov. *Int J Syst Evol Microbiol* 54(Pt 3)**,** 689-691. doi: 10.1099/ijs.0.02888-0.

Viallard, V., Poirier, I., Cournoyer, B., Haurat, J., Wiebkin, S., Ophel-Keller, K., et al. (1998). *Burkholderia graminis* sp. nov., a rhizospheric *Burkholderia* species, and reassessment of [*Pseudomonas*] *phenazinium*, [*Pseudomonas*] *pyrrocinia* and [*Pseudomonas*] *glathei* as *Burkholderia*. *Int J Syst Bacteriol* 48 Pt 2**,** 549-563. doi: 10.1099/00207713-48-2-549.

Yuan, Z.C., Zaheer, R., Morton, R., and Finan, T.M. (2006). Genome prediction of PhoB regulated promoters in *Sinorhizobium meliloti* and twelve proteobacteria. *Nucleic Acids Res* 34(9)**,** 2686-2697. doi: 10.1093/nar/gkl365.
